# Peripheral nerve defects repaired with autogenous vein grafts filled with platelet-rich plasma and active nerve microtissues and evaluated by novel multimodal ultrasound techniques

**DOI:** 10.1186/s40824-022-00264-8

**Published:** 2022-06-11

**Authors:** Yaqiong Zhu, Nan Peng, Jing Wang, Zhuang Jin, Lianhua Zhu, Yu Wang, Siming Chen, Yongqiang Hu, Tieyuan Zhang, Qing Song, Fang Xie, Lin Yan, Yingying Li, Jing Xiao, Xinyang Li, Bo Jiang, Jiang Peng, Yuexiang Wang, Yukun Luo

**Affiliations:** 1 Departments of Ultrasound, The First Center of Chinese PLA General Hospital, Beijing, China; 2 Beijing Key Lab of Regenerative Medicine in Orthopedics, Chinese PLA General Hospital, Beijing, China; 3 Key Lab of Musculoskeletal Trauma & War Injuries, Chinese PLA General Hospital, Beijing, China; 4 Beijing Key Laboratory of Chronic Heart Failure Precision Medicine, Chinese PLA General Hospital, Beijing, China; 5 Department of Geriatric Rehabilitation, The Second Center of Chinese PLA General Hospital, Beijing, China; 6 Department of Orthopedic Surgery, The First Affiliated Hospital of University of Science and Technology of China, Hefei, Anhui Province China; 7 General hospital of Northern Theater Command, Liaoning, China; 8 Department of Anesthesiology, JiangXi PingXiang People’s Hospital, Jiangxi, China

**Keywords:** Platelet-rich plasma, Microtissues, Autogenous vein, Multimodality ultrasound, Nerve defect

## Abstract

**Background:**

Developing biocompatible nerve conduits that accelerate peripheral nerve regeneration, lengthening and functional recovery remains a challenge. The combined application of nerve microtissues and platelet-rich plasma (PRP) provides abundant Schwann cells (SCs) and various natural growth factors and can compensate for the deficiency of SCs in the nerve bridge, as well as the limitations of applying a single type of growth factor. Multimodal ultrasound evaluation can provide additional information on the stiffness and microvascular flow perfusion of the tissue. This study was designed to investigate the effectiveness of a novel tissue-engineered nerve graft composed of an autogenous vein, nerve microtissues and PRP in reconstructing a 12-mm tibial nerve defect and to explore the value of multimodal ultrasound techniques in evaluating the prognosis of nerve repair.

**Methods:**

In vitro, nerve microtissue activity was first investigated, and the effects on SC proliferation, migration, factor secretion, and axonal regeneration of dorsal root ganglia (DRG) were evaluated by coculture with nerve microtissues and PRP. In vivo, seventy-five rabbits were equally and randomly divided into Hollow, PRP, Micro-T (Microtissues), Micro-T + PRP and Autograft groups. By analysing the neurological function, electrophysiological recovery, and the comparative results of multimodal ultrasound and histological evaluation, we investigated the effect of these new nerve grafts in repairing tibial nerve defects.

**Results:**

Our results showed that the combined application of nerve microtissues and PRP could significantly promote the proliferation, secretion and migration of SCs and the regeneration of axons in the early stage. The Micro-T + PRP group and Autograft groups exhibited the best nerve repair 12 weeks postoperatively. In addition, the changes in target tissue stiffness and microvascular perfusion on multimodal ultrasound (shear wave elastography; contrast-enhanced ultrasonography; Angio PlaneWave UltrasenSitive, AngioPLUS) were significantly correlated with the histological results, such as collagen area percentage and VEGF expression, respectively.

**Conclusion:**

Our novel tissue-engineered nerve graft shows excellent efficacy in repairing 12-mm defects of the tibial nerve in rabbits. Moreover, multimodal ultrasound may provide a clinical reference for prognosis by quantitatively evaluating the stiffness and microvescular flow of nerve grafts and targeted muscles.

## Background

Peripheral nerve injuries are common in both civilian and military environments and are primarily transection injuries, that can occur during motor vehicle accidents, sports activities, surgery or other forms of penetrating trauma [1]. An end-to-end suture is the preferable technique for nerve repair. However, injuries may result in extensive nerve gaps that do not allow for primary repair; in such cases, the defect should be repaired with a nerve graft of appropriate size. Autogenous nerve grafting is currently the procedure of choice for tensionless repair of extensive nerve grafts, and this procedure provides the best regeneration results [2]. The nerve to be grafted is often harvested from the sural and medial and lateral antebrachial cutaneous nerves [3]. Unfortunately, there is a limited supply of nerves for grafting and significant donor-site morbidity associated with harvesting, such as the need for a second surgical step and elimination of donor nerve function; additionally, in some cases, there may be a mismatch between nerve and graft dimensions [3]. Problems with autogenous nerve grafting have led to extensive research into alternative methods of repair. A variety of techniques, including veins and synthetic grafts, have been developed. Our experiment utilized a vein conduit for nerve regeneration. The tubulization technique is an alternative repair method that consists of suturing the nerve stumps into a tubular guide to offer mechanical guidance as well as an optimal environment for advancing axonal sprouts.

Autologous veins are biological materials that are readily harvested for donor conduits of a variety of sizes and lengths; they are rich in laminin, and most importantly, they are nonimmunogenic [4]. The walls of veins are thin and resilient, substantial enough to act as barriers against ingrowth of connective tissue but permeable enough to allow diffusion of the proper nutrients for nerve regeneration [5]. Unlike artery and nerve donor sites, vein donor sites carry a low risk for morbidity resulting from the harvesting procedure, and there are minimal consequences from loss of the donor structure [5]. Vein tubes have been validated for clinical application as autogenous nerve conduits with improved outcomes over time [6–8]. Chiu [6] et al. observed uniformly significant symptom relief and satisfactory sensory function recovery in patients when autogenous vein grafts were used as nerve grafts in segmental nerve injuries of 3 cm or less. The same author published a review [7] reaffirming the effectiveness of autogenous venous tubes as a vehicle for modulating the cellular and molecular environment for nerve regeneration.

Vein wall collapse due to tissue compression is a potential problem. It has been reported that this can be circumvented by filling the vein with muscle tissue, extracellular matrix components, tendons and stem cells [9]. Autologous nerve microtissue, or autologous minced nerve tissue, is a possible vein graft filler material that can be derived from damaged tissue removed from the injury site; thus, in nerves of minor importance, large defects can be repaired with this technique. Autologous nerve microtissues appear as fine particles and contain amount of Schwann cells (SCs), which can not only support veins but also proliferate and secrete various neurotrophic factors in the vein to promote axon regeneration due to the favourable biological characteristics veins [10, 11].

Platelet-rich plasma (PRP) is another possible filler material for vein grafts [12]. PRP is an autologous concentrate of platelets in a small volume of plasma containing growth factors released by these cells, which can serve as a matrix for enhancing collagen synthesis, stimulating angiogenesis, accelerating SC migration, and promoting nerve regeneration [13, 14]. Once activated by thrombin, PRP filling inside the vein can provide good mechanical support, which does not easily leak out and exerts its effects for a long period [15]. Recent studies also reported that PRP had positive effects on nerve regeneration, in addition to its positive effects on the healing of many types of tissues [16, 17].

Multimodal ultrasound has proven to be a valuable tool in traumatic nerve injuries [18, 19]. Ultrasound can reliably distinguish (fascicular) perineurium from interfascicular epineurium in humans and can be used to identify and localize disruption of nerve continuity and other gross structural anomalies. Shear-wave elastography (SWE) and contrast-enhanced ultrasonography (CEUS) can complement conventional ultrasound and help to represent the mechanical characteristics and subtle blood flow changes in peripheral nerves [19]. In recent years, AngioPLUS (PlaneWave UltraSensitive Imaging; Supersonic Imagine, Aix-en-Provence, France) technology has overcome the limitations of traditional colour Doppler ultrasound in microvascular imaging performance [18]. Recently, published studies have shown that this technique is highly sensitive to the detection of microflow inside malignant breast nodules, which helps to improve the accuracy of the diagnosis of benign and malignant breast nodules [18]. However, there are few studies on peripheral nerve injury using multimodal ultrasound.

In the present study, we designed a novel style of tissue-engineered nerve graft composed of a vein, nerve microtissues and PRP, all of which are autogenous tissues. The aim of this study was to evaluate the role of new nerve grafts in the reconstruction of peripheral nerve defects. The value of multimodal ultrasound techniques (conventional ultrasound, SWE, CEUS, and AngioPLUS) in evaluating the prognosis of nerve repair was also explored by comparing the results of multimodal ultrasound and histopathological analysis.

## Materials and methods

The study design included in vitro and in vivo studies (Tables 1 and 2). All procedures described in this study were approved by the Ethics Committee of the Chinese PLA General Hospital (No. 2016-× 9–07). All experiments were performed at the Beijing Key Laboratory of Regenerative Medicine in Orthopaedics, Chinese PLA General Hospital, Beijing, China.

**Table 1:** Framework for in vitro study

	Treatment
In vitro study	PRP preparation
	Active nerve microtissue identification
	Neurotrophic factors identification
	SCs co-cultured with nerve microtissues and/or PRP
	DRGs co-cultured with nerve microtissues and/or PRP
	SCs migration study

**Table 2:** In vivo study at different time point

In vivo study (time point)	Treatment	
	Vein graft	Target muscle (Triceps surae muscle)
2 weeks	High-frequency ultrasound (*n* = 8 animals/group)	–
	CEUS (*n* = 3 animals/group)	
	qRT-PCR (VEGF) (*n* = 3 animals/group)	
4 weeks (*n* = 4 animals/group)	H&E staining	–
	Immunofluorescence staining (S100, NF200)	
12 weeks	Nerve function (*n* = 8 animals/group)	High-frequency ultrasound (*n* = 8 animals/group)
	High-frequency ultrasound (*n* = 8 animals/group)	SWE and AngioPLUS examinations (*n* = 8 animals/group)
	Macroscopic evaluation (*n* = 8 animals/group)	CEUS (*n* = 5 animals/group)
	Electrophysiological recovery evaluation (*n* = 5 animals/group)	Macroscopic evaluation (*n* = 5 animals/group)
	Morphometrical evaluation (Electron microscope) (*n* = 5 animals/group)	Morphometrical evaluation (H&E, Masson staining) (*n* = 5 animals/group)

### In vitro study

#### Preparation of PRP

**Fig. 1:**
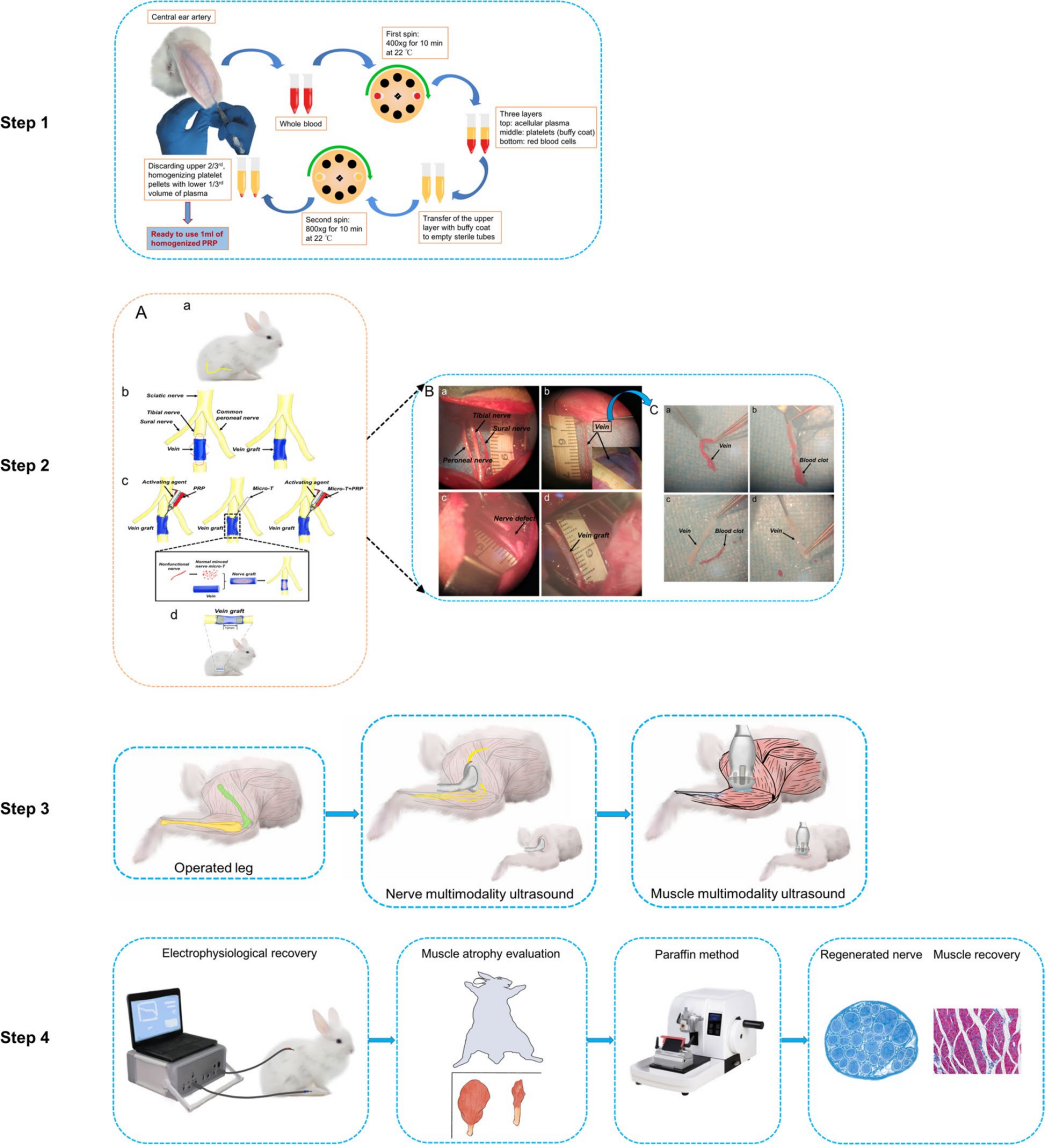
Illustration of the comprehensive steps in vivo study. (I) Step1 The preparation of autogenous PRP. (II) Step 2 The detailed procedure of repair of 12 mm tibial nerve defect with vein graft. **A** Schematic illustration of autogenous vein graft filling of PRP, nerve microtissues, and a combination of the two. An autogenous vein graft was transplanted to the tibial nerve to repair the 12 mm nerve defect. A 18-mm-long autogenous vein graft was harvested from the superficial subcutaneous vein at the surgical approach below the right femur (**B**). The Figure **C** showed the blood clot inside the vein was removed by rinsing in saline solution. (III) Step 3 Multimodality ultrasound evaluation of nerve graft and innervated calf muscle. (IV) Step 4 Evaluation of electrophysiological recovery and histopathological examination of regenerated nerve and target muscle

PRP was prepared by the method previously reported [20] (Fig. 1, step 1). A total of 8 mL whole blood of rat was collected via cardiac puncture and placed in a 10 ml sterile tube containing 3.8% w/v sodium citrate. The collected blood was immediately centrifuged at 400 g for 10 minutes. Following centrifugation, three layers were formed: the upper layer, that was acellular plasma; middle layer, containing abundant platelets; and bottom layer, with rich red blood cells. The supernatant and a small portion of the transition zone (buffy coat) were carefully transferred into another non-anticoagulant sterile tube and centrifuged at 800 g for 10 minutes, yielding approximately 1 ml of PRP. For quality testing, PRP sample and whole blood sample were sent to the laboratory with 200 μl each. The number of platelets, red blood cells, and white blood cells was counted. Further experiments were performed when the platelet concentration in PRP was 4 ~ 6 times of that in whole blood and the amount of erythrocytes and leukocytes was extremely low.

PRP was activated with a mixture of bovine thrombin (Sigma, T4648) and 10% calcium chloride (Sigma, c1016) solution. After a few seconds, the PRP clot was formed, allowing the clot to retract for about 15 min to release enough growth factors. Next, the supernatant containing a large amount of growth factors was obtained by centrifugation at 2800 g for 10 minutes. The supernatant was stored in a refrigerator at − 80 °C until it was used to supplement the culture medium for further experiments.

#### Preparation and identification of active nerve microtissues

Nerve microtissues were harvested from the sciatic nerves of 3-day-old Sprague–Dawley (SD) rats (Vital River Laboratory Animal Technology Co., Ltd., Beijing, China; license number SCXK (Jing) 2016–0006) according to established method [21]. Briefly, after the 15 rats were sacrificed and sterilized, the sciatic nerves were exposed and dissected through longitudinal surgical incisions behind the femurs on both sides. The epineuria of sciatic nerves were first removed and then the nerves were minced into 1 mm × 1 mm fine particles (nerve microtissues). Nerve microtissues were cultured in DMEM/F-12 (Gibco, USA) containing 10% (v/v) fetal bovine serum (FBS; Gibco), 1% penicillin–streptomycin solution (Gibco), 10 ng/ml heregulin-β1 (Sigma-Aldrich) and 2 mM forskolin (Sigma-Aldrich) in five six-well culture plates. Then, this was followed by incubation in an atmosphere of 5% CO_2_ at 37 °C for 1, 3, 5, 7, and 10 days. Culture medium were collected and stored in a refrigerator at − 80 °C at each time point for further testing. The live nerve microtissues were identified by FDA/PI bichromatic fluorescence staining and the SCs derived from microtissues were identified by S-100 immunofluorescence staining at each time point. In addition, SCs in each well were enzymatically hydrolyzed with 300 ul 0.2% (w/v) collagenase NB4 (Sigma-Aldrich) for 1 min. The mixture of DMEM/F-12 mixture containing 10% FBS was used to terminate enzymatic hydrolysis reaction, and then counted the SCs numbers.

#### Determination of neurotrophic factors secreted by nerve microtissues

The collected culture mediums at 1, 3, 5, 7, and 10 days were used for determining the concentration of the neurotrophic factors secreted by live nerve microtissues. The concentrations of nerve growth factor-β (NGF-β), vascular endothelial growth factor (VEGF), brain-derived neurotrophic factor (BDNF) and glial cell line-derived neurotrophic factor (GDNF) were determined using an NGF-β ELISA kit (Boster, EK0471), a VEGF ELISA kit (Boster, EK0540), a BDNF ELISA kit (Boster, 0308) and a GDNF ELISA kit (Boster, EK0363) as instructed by the manufacturer.

#### Schwann cells were co-cultured with nerve microtissues and/or PRP supernatant

Microtissues and PRP supernatant were harvested following the procedures described above. Schwann cells were harvested as follows. Briefly, the sciatic nerves with the epineuria removed and were minced from 10 SD rats of 3-day-old and then enzymatically dissociated with 1 ml of 0.2% (w/v) collagenase NB_4_ (Sigma-Aldrich) for about 10 min in a 37 °C incubator and stirred with a magnetic stirrer. After that, the mixture was centrifuged for 5 min with 1500 rpm/min and resuspended in SCs medium and transferred into a 25 cm^2^ cell culture flask that was incubated in an atmosphere of 5% CO_2_ at 37 °C. The third passage (P3) cells were utilized in this study.

The SCs were placed in the lower chamber of the twelve-well transwell co-culture system. A total of 8 × 10^4^ cells were seeded on the cell slide in each well, maintaining with 2 ml of serum DMEM/F-12 medium for 24 h and allowed to attach overnight. Subsequently, SCs were co-cultured with nerve microtissues and/or PRP supernatant. SCs medium was added to the upper chamber as control group. The nerve micro-tissues, PRP supernatant and the mixture of nerve microtissues and PRP supernatant served as corresponding three experiment groups were added to each upper compartment in quadruplicate. After co-cultured for 1, 3, 5 and 7 days, one of the wells in each group was enzymatically hydrolyzed with 200ul 0.2% (w/v) collagenase NB4 (Sigma-Aldrich) for 1 min, and then DMEM/F-12 mixture containing 10% FBS was added to terminate enzymatic hydrolysis reaction. Finally, the cell count was performed.

At the different time point, the 2 ml DMEM/F-12 medium and 200 μl of Cell Counting Kit-8 (CCK-8, Dojindo Laboratories, Shanghai) colorimetric assay reagent were added to each well to evaluate the effect of co-culture on SCs proliferation. The optical density (OD) value was measured at 450 nm by using a microplate reader (Epoch, Biotek, US). The absorbance was directly proportional to the number of living cells. Furthermore, the morphology and distribution of SCs in different groups were detected by S-100 immunofluorescence staining.

#### Dorsal root ganglions (DRGs) were co-cultured with microtissues and/or PRP supernatant

The DRGs were extracted from three 12-h-old SD rats. Briefly, the SD rats were firstly sacrificed and sterilized by soaking in 75% ethanol solution. The SD rat was placed in a prone position, then the skin was cut open along the midline of the back, and the blood was washed with DMEM/F-12 after the spine was completely removed. The spine was then divided into two halves with microscissors along the midsagittal segment under the microscope to expose the bilateral intervertebral foramen, and the DRG was removed completely. The epineurium of DRG was carefully stripped in the frozen DMEM/F-12 medium supplemented with 10% FBS, and care was taken not to clamp the body of DRG. Finally, DRGs were inoculated on the cover glass of the lower chamber of the twelve-well transwell co-culture system, and an appropriate amount of DRG culture medium was added. The DRG medium, PRP supernatant, nerve microtissues and the mixture of nerve microtissues and PRP supernatant were added in the upper chamber of transwell co-culture system, respectively. They were placed and cultured in an incubator at 37 °Cwith 5% CO2. After 5 days, the insert was carefully removed, and the DRGs were fixed with 4% paraformaldehyde. The regenerative capacity of axons was detected by S-100 and NF-200 immunofluorescence staining. Five DRGs were randomly selected from each group and were integrally photographed. Each DRG image was divided into four quadrants, and the longest five axons in each quadrant were measured. Image Pro Plus 6.0 (IPP 6.0, Media Cybernetics, USA) image analysis software was used to calculate the average maximum axon length of each DRG.

#### Schwann cells migration study

In order to test the capacity of the PRP supernatant, neurotrophic factors secreted by nerve microtissues, and a combination of two to induce SCs migration, six-well transwell systems with 8 μm pores (Corning Costar, USA) were used in this study. A total of 1.5 × 10^4^ SCs were added in each of the upper chamber. Serum medium (FBS group), serum medium with 500 μl PRP supernatant (PRP group), 5-day nerve microtissues medium 500 μl (Micro-T group), or medium with the latter two samples (Micro-T + PRP group) were added to the lower chambers. After incubation in a humidified atmosphere (37 °C, 5% CO_2_) for 12 h, the upper surface of each membrane was cleaned with a cotton swab. The migrated SCs adhered to the underside of the membrane, which were fixed with 4% paraformaldehyde and stained by crystal violet solution. Five randomly selected visual fields (200 x magnification) were captured from each slide to calculate the number of migrated SCs using Image Pro Plus 6.0 software. The migration ratio of SCs refers to the ratio between the number of SCs migrated in each experimental group and the number of SCs migrated in FBS group.

### In vivo study

#### Animals

Seventy-five 4-month-old healthy, male and clean New Zealand white rabbits weighing 2.5 to 3.0 kg were provided by the Animal Breeding Centre of Long’ an, Beijing, China (licence no. SCXK [Jing] 2014–0003). The rabbits were housed individually in cages at room temperature with a 12-hour light/dark cycle. They were received food and water ad libitum. The rabbits were randomly divided into five groups with 15 rabbits in each group. The groups included (1) a Hollow group in which the defect of tibial nerve was repaired using a autogenous vein with saline infusion; (2) a PRP group, autogenous vein was filled with autogenous PRP to repair the transection injury of tibial nerve; (3) a nerve Micro-tissue (Micro-T) group, autogenous vein was filled with autogenous tibial nerve microtissues to repair the transection injury of tibial nerve; (4) a Micro-T + PRP group, autogenous vein was filled with the mixture of autogenous tibial nerve micro-tissues and PRP to repair the transection injury of tibial nerve; (5) an Autograft group, the defect of tibial nerve was repaired using an excised autogenous nerve graft from the same locale.

#### Autologous PRP preparation

Before surgery, 8 ml of whole blood was extracted from the central ear artery of rabbits in the PRP and Micro-T + PRP groups. The preparation procedures of PRP was the same in vitro study. The prepared PRP (1 ml) was stored in a − 80 °C refrigerator for use during surgery (Fig. 1, step 1).

#### Surgery protocol

Rabbits were first weighed and then intramuscular injected with 3% pentobarbital sodium solution (1 ml / kg) for anesthsia. The right lower extremity of all animals was shaved and disinfected with iodine solution. All surgical procedures were performed under aseptic operating conditions by two surgeons. A 18-mm-long autogenous vein graft was harvested from the superficial subcutaneous vein at the surgical approach below the right femur. The blood clot inside the vein was removed by rinsing in saline solution. The vein was then stored temporarily in normal saline. Next, three major fascicles (tibial nerve, common peroneal nerve and sural nerve) of the right sciatic nerve were exposed by a gluteal muscle-splitting incision that was between the vastus lateralis and the biceps femoris muscles. A 12-mm-long tibial nerve was excised at the midthigh and a 12-mm-long gap was created. Since the vein was retracted after harvest, the vein that was excised is slightly longer than the length of the nerve defect. The distal and proximal stumps of the autologous vein were reversed and sutured to both ends of the tibial nerve, and 1 mm of each nerve stump was inserted into the vein graft. Every effort was made to avoid tension and keep correct rotational alignment throughout (Fig. 1, step 2).

##### Hollow group

The tibial nerve defect was repaired only by the autologous vein graft with saline infusion, avoiding the collapse of the vein wall.

##### PRP group

The collected PRP was taken out from a − 80 °C refrigerator and restored to room temperature. PRP and activator were loaded into a double syringe. The 300 μl PRP was simultaneously injected into the veingraft with the 300 μl activator (the mixture of 10% calcium chloride (Sigma, c1016) solution and 1000 units of bovine thrombin (Sigma, T4648)). The PRP was activated and formed a PRP gel in the vein to avoid leakage (Fig. 1, Step 2).

##### Micro-T group

After the excision of a 12-mm long tibial nerve, it was divided into three equal parts (4-mm/part), one of which was stripped of the epineurium and cut into nerve microtissues under sterile conditions. The minced nerve microtissues were distributed equally in the lumen of the vein graft.

##### Micro-T + PRP group

The 4-mm length of microtissues stripped of the epimembrane was mixed with 300 μl PRP and injected into the vein simultaneously with the activator. The mixture was activated in the vein to form a gel that avoided leakage of microtissues.

##### Autograft group

The excised tibial nerve was reversed and sutured to both ends of the tibial nerve to repair the defect from the same locale.

After the animal model was established, the muscular layer and skin were sutured with 3–0 monofilament nylon and disinfected with iodine solution.

## Postoperative care

Postoperatively, all rabbits were provided free access to water and standard rabbit nutrients. Animals were examined twice daily for 10 days to check for wound healing and infection, and were recorded in the laboratory records. Postsurgical infection was controlled by injection of antibiotics (800,000 IU of penicillin daily) intramuscularly for 5 days. The animals were monitored by a specialist veterinarian under standard laboratory conditions during the 3-month postoperative period.

## Nerve recovery assessment

### Nerve function evaluation

The sciatic nerve function was evaluated at 12 weeks. The toe spreading score (graded from 1 to 4 points) [22] and the modified Tarlov score (rated from 0 to 4 points) [23] were used to evaluate nerve function. Higher scores indicated better nerve function recovery.

### High-frequency ultrasound and contrast-enhanced ultrasonography (CEUS) examination of vein grafts

All of the ultrasound procedures were performed by a radiologist with nine years of ultrasound experience. All machine settings, such as depth, gain and focus, were kept constant during each measurement. The thickness of vein grafts was examined at 2 weeks and 12 weeks after operation and the perfusion of vein grafts was examined at 2 weeks after operation. In short, after the animals were anesthetized, the vein grafts were scanned longitudinally using a high-frequency ultrasonic equipment (Vevo 3100, Visualsonics, Canada) with a 14- to 28- MHz linear array probe (MX250). The probe was then rotated 90° to measure the thickness of the vein grafts in a cross section. The mean value of 3 measurements was taken for statistical analysis (Fig. 1, step 3).

Ultrasonic contrast agent is pure blood pool contrast agent, has a unique advantage for tissue microcirculation imaging. In this study, the CEUS examination, a method for detecting the regenerated microvessels and their perfusion in the vein grafts, was performed using a high-resolution ultrasound system (Mindray, Resona 7) at a low mechanical index (MI 0.05–0.07). This device was equipped with a linear array transducer (4–15 MHz). The procedures of CEUS examination was the same as previously reported [19]. SonoVue (Bracco International, Milan, Italy), a sulfur-hexafluoride-filled microbubble contrast agent, which was encapsulated by a flexible phospholipid shell. It is very safe for use in animals or humans because adverse allergic reactions are rare, and it can be exhaled within 15 minutes after intravenous injection. At 2 weeks after the operation, SonoVue (mixed with 5 ml of saline) was injected into a peripheral ear vein at a bolus of 0.13 ml/kg, followed by a 2 ml saline flush for the CEUS examination. Dynamic ultrasound videos stored continuously for at least 60s were used to analyze the time to peak (TTP), peak intensity (PI) and area under the curve (AUC) of region of interest (ROI), which were all parameters reflecting blood perfusion in early postoperative vein grafts. The mean results of 3 repeated analyses of a vedio were presented.

### Quantitative real-time RT-PCR (qRT-PCR)

At 2 weeks following the treatment, total RNA of vein/nerve grafts was isolated using RNA extraction solution (Servicebio, G3013). The FastStart Universal SYBR Green Master (Rox) (Servicebio, G3008) was used to assess VEGF gene expression. The glyceraldehyde-3-phosphate-dehydrogenase (GAPDH) served as reference household gene used to normalize the amount of mRNA. Then the sequence of the primers was selected and carefully checked as follows: VEGF forward 5′-GTCCTCAAAGCATCAGCATAAGAA-3′, reverse 5′--3′; GAPDH, forward 5′- CTGGAGAAACCTGCCAAGTATG-3′, reverse 5′- GGTGGAAGAATGGGAGTTGCT-3′. Relative changes in gene expression were calculated using the comparative Δ crossover threshold (CT) method.

### Macroscopic evaluation of vein graft adherence

The nerve repair sites in animals from each group were evaluated at 12 weeks at the end of ultrasound follow-up with the animals under deep anesthesia. The assessment of vein graft adherence was performed blindly and followed an established numeric grade scheme [24]. The degree of venous graft adherence was divided into three levels. No dissection or mild blunt dissection (grade I); some vigorous blunt dissection required (grade II); and sharp dissection required (grade III) [24].

### Electrophysiological recovery evaluation

A fully functional electromyography (EGM) machine (Keypoint, Medtronic) was used for the electrophysiological evaluation. At 12 weeks, after macroscopic evaluation of vein graft, electrophysiological tests were performed to assess the regeneration of myelinated nerve fibers. After a moderate dose of anaesthetic was administered to the rabbit, the tibial nerve operating area in the right thigh, the tibial nerve in the healthy side and the bilateral triceps surae muscles were exposed. The two stimulation electrodes (6 Hz, 5 mA) were placed in order at the proximal and distal ends of the vein grafts, and the monopolar recording electrode was placed on the belly of the triceps surae muscle of the same side to induce and record the compound muscle action potentials (CMAPs). The CMAPs of the normal tibial nerve also need to be detected and recorded. The amplitude and latency of bilateral CMAPs were recorded 5 times for each animal, and the analysis results were expressed as the ratio of the injury side to the normal side (Fig. 1, step 4).

### Histological evaluation of regenerated nerves at early stage

Animals were sacrificed by overdose injection of a sodium pentobarbital at 4 weeks after vein transplantation. The implanted vein grafts were isolated from the surrounding tissue and divided into three parts after the integral excise. The middle and distal transversal segment of grafts was rapidly fixed in 4% paraformaldehyde for 2 hours and then embedded in paraffin. The 4 μm thick transversal sections were obtained H&E staining and immunofluorescence staining.

Commercial H&E staining kit (G1120, Solarbio) was used for H&E staining, followed by a series of routine dehydration, transparency and sealing steps. As for immunofluorescence staining, the sections of each vein graft were blocked at room temperature with 10% goat serum for 1 hour after washing three times (5 min/time) each time with PBS. Rabbit anti-S100 antibody (1:200, bs-2015R, Bioss) and mouse anti-Neurofilament 200 antibody (1:200, N5389, Sigma) were, respectively, applied as the primary antibodies and incubated in a humidified chamber overnight at 4 °C. Next morning, the remaining liquid was removed from the sections and washed three times in PBS. Then, the sections were incubated with goat anti-rabbit IgG H&L (Alexa Fluor 488, 1:200, ab150077, Abcam) and goat anti-mouse IgG H&L (Alexa Fluor 594, 1:200, ab150116, Abcam) secondary antibody in the dark for 2 hours at room temperature. After washing with PBS for three times (5 min/time), the nuclei of SCs were counterstained with DAPI (1:200). A fluorescence microscope (200× magnification, Nikon Eclipse C1, Japan) and IPP 6.0 software were used for capturing images (5 random fields were selected from each slice) and analyzing the mean density of regenerated axons of each group at 4 weeks, respectively.

### Morphometrical assessment of regenerated nerves

The semithin sections and ultrathin sections were acquired to evaluate the morphology of regenerative nerves (fiber diameter, and myelin sheath thickness). At 12 weeks, animals in each group were randomly sacrificed and 5 mm length of regenerative nerves at the end of distal vein grafts were removed. The excised nerves were rapidly fixed in precooled 2.5% (w/v) glutaraldehyde for 3 hours, then in 1% (w/v) osmic acid solution for 1 hour, finally washed, dehydrated through a series of grades of ethanol solutions, and embedded in epoxy resins. The nerve segments were cut into 1.5 μm-thick semithin slices with a glass knife and 70 nm-thick ultrathin slices with a diamond knife.

The semithin sections were stained with Toluidine Blue solution (1% in sodium borate, G3663, Solarbio), and then five fields at 400x magnification were randomly selected for each animal. The mean density of myelinated nerve fibers was counted by IPP 6.0 software. Furthermore, the morphology of myelin sheath, diameter of fiber, and myelin sheath thickness were observed in ultrathin sections and analysed by IPP 6.0 software.

## Triceps surae muscle recovery assessment

### Multimodal ultrasound evaluation

#### High-frequency ultrasound evaluation

At 12 weeks, high-frequency ultrasound equipment with a 21- to 44- MHz linear array probe (MX4000, Vevo 3100, Visualsonics, Canada) was used for assessing echogenicity and thickness difference (measured in maximal cross-sectional area, CSA) of innervated targeted muscle (triceps surae muscle) in each group. The mean value of 3 measurements was taken for statistical analysis.

#### Shear wave elastography (SWE) and Angio PlaneWave UltrasenSitive (AngioPLUS) imaging evaluation

At 12 weeks, the stiffness and the microvascular flow of the triceps surae muscle were detected and measured with the Aixplorer system (Supersonic Imagine, Aix-en-Provence, array transducer Super Linear L15–4, France) equipped with SWE and AngioPLUS tecniques. Different from other ultrasound techniques, the images displayed by SWE and AngioPLUS tecniques can be diaplayed simultaneously on the screen, so that the relationship between the degree of the target muscle fiber tissue hyperplasia, that is, the stiffness variation and the changes of microvascular flow density in the muscle can be obtained.

Double image mode was adopted for detection. The ROI area (a circular area of 10 mm in diameter) was selected at the mid-belly of the triceps surae muscle in a longitudinal plane of ultrasound to measure the stiffness and microvascular flow density of the muscle at the same time. The image was frozen when the map was stable in 3–5 s. The mean Young’s modulus values were averaged for three measurements. The interval between every two measurements should be at least 5 s. In addition, the blood flow frequency spectrum was used to confirm that the images presented were microvessels but not artifacts. The value of Young’s modulus represents the stiffness. The amount of microvascular flow density (total area of microvascular flow/ ROI area) was analyzed by IPP 6.0 software, and the mean value of three measurements were taken in each case for the statistical analysis. The monochrome pattern of the microvascular muscle was also displayed. Finally, the correlation analysis was performed between Young’s modulus values and microvascular flow density.

#### CEUS evaluation

CEUS examination of targeted muscles was performed at 12 weeks after the operation, and the examination method was similar to that of vein grafts. The video of each animal was analyzed three times and averaged for final statistical analysis.

### Macroscopic evaluation

At 12 weeks, after multimodality ultrasound examination, the animals were sacrificed in each group. Bilateral triceps surae muscle were removed and weighed immediately. The wet weight recovery rate of the muscle was the ratio of the wet weight of the muscle from operative side to that of the normal side.

### Morphological evaluation

At 12 weeks after surgery, the H&E and Masson’s trichrome staining were performed for detecting the morphology of the muscle. In other words, H&E staining was used to observe adipocytes infiltration and nuclear distribution in muscle, and Masson’s trichrome staining was used to evaluate collagen proliferation in muscle tissue and atrophy recovery of muscle fibers. The triceps surae muscles in each group were harvested and fixed in 4% paraformaldehyde for 2 hours and then embedded in paraffin. The 4 μm thick transversal sections were obtained. The method of H&E staining was the same as that of nerve tissue, and the Masson’s trichrome staining was performed by using a modified Masson’s trichrome stain kit (G1345, Solarbio). Five randomly selected fields in each slide at 200x magnification were captured to measure the average area of muscle fibers and positive area percentage of collagen with IPP 6.0 software. The mean value of each slide was used for the final statistical analysis.

## Statistical analysis

Statistical analyses were performed using Statistical Program for Social Sciences (SPSS) software (version 22.0) and GraphPad 8.0 (Graphpad Software, Inc. San Diego, CA, Unit). The Kolmogorov-Smirnov test was used for a normal distribution test of the data. If the data was normally distributed and the variance was uniform, the differences between two groups were compared by Student’s t-test and the multiple comparisons were tested by one-way ANOVA analysis. Tukey’s multiple comparison post hoc test was applied when *P* > 0.05 in the test of homogeneity of variances; otherwise, Dunnett’s T3 post hoc test was applied. Pearson’s correlation analysis was performed to analyze the correlation between the two variables. Statistically significant was defined as *P* < 0.05 between groups. According to the post hoc power analysis, a power of the main indicators of each group was > 80% at 12 weeks after the operation, with a significance level of 0.05, indicating that no additional animals were needed.

## Results

### In vitro study

#### Blood cell test of PRP

The concentration of the platelets in prepared PRP was 1749.85 ± 331.44 × 10^3^ platelets/μL, which was approximately 4.8 times the concentration in whole blood. In addition, the concentrations of red blood cells (RBCs) and white blood cells (WBCs) in PRP were 2.03 ± 0.57 × 10^9^ /mL (5.1–7.6 × 10^9^ /mL) and 2.14 ± 0.91 × 10^6^ /mL (5.2–12.5 × 10^6^ /mL), respectively, which were significantly lower than those in whole blood.

#### Identification of active nerve microtissues

**Fig. 2:**
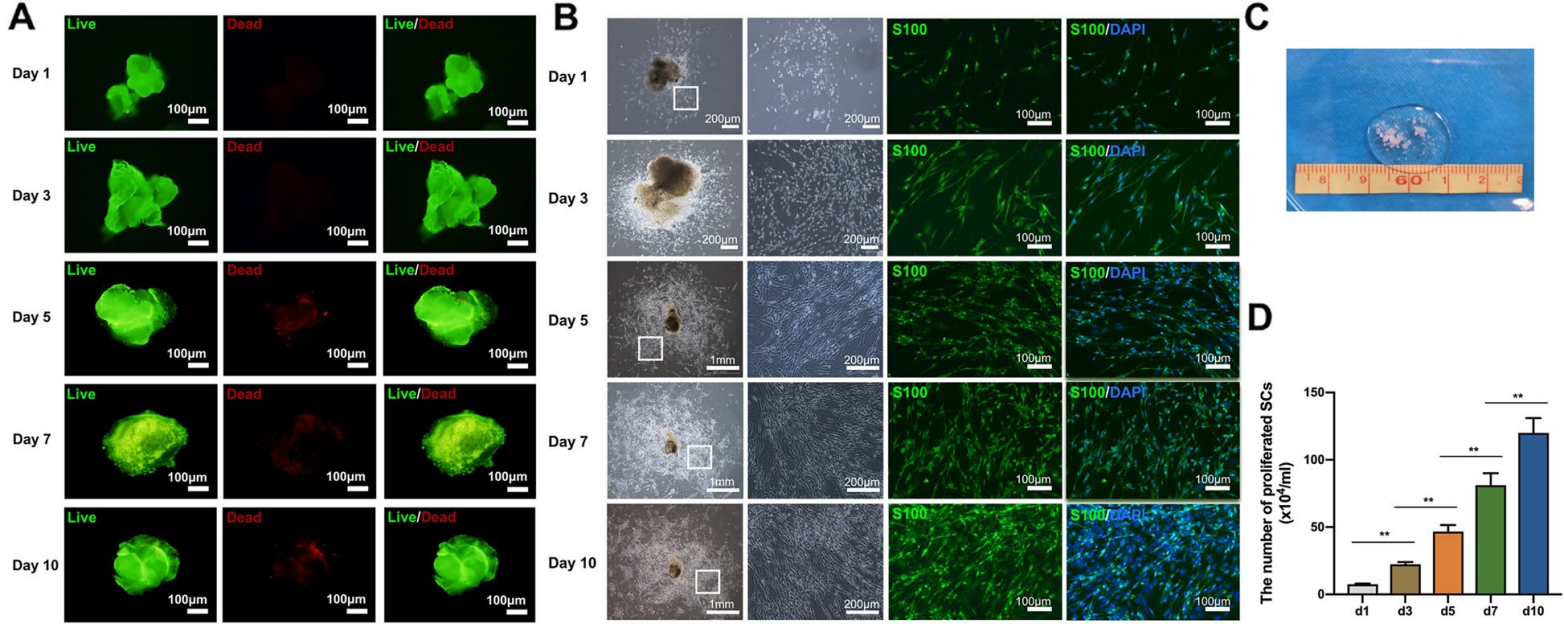
Identification of living nerve microtissues. **A** FDA/PI bichromatic fluorescence staining of live nerve microtissues after 1, 3, 5, 7, and 10 days of culture. Green fluorescence denotes living cells and red fluorescence denotes dead cells. **B** Anti-S-100 immunofluorescence staining of cells proliferating around the microtissues, showed a large number of SCs. **C** A general view of the chopped microtissues (1 mm ×1 mm fine particles). **D** The SCs count during 1, 3, 5, 7, and 10 days of culture (^**^P < 0.01). SCs Schwann cells

After 1, 3, 5, 7, and 10 days of culture in the incubator, the fluorescein/propidium iodide (FDA/PI) bichromatic fluorescence staining showed that the nerve microtissues were still alive over time, showing bright green fluorescence, while only a few dead cells showed dark red fluorescence (Fig. 2A).

In addition, as shown in Fig. 2B, numerous proliferating cells appeared around the nerve microtissue over time (1, 3, 5, 7, 10 days) during in vitro culture, while the nerve microtissue itself gradually dissolved and decreased. Furthermore, the anti-S-100 immunofluorescence staining results showed that the cells proliferating around the microtissue were positively stained, confirming that the microtissue could be decomposed into SCs in vitro (Fig. 2B). Figure 2C presents a general view of the chopped microtissues. Figure 2D displays the number of SCs corresponding to different days of culture of nerve microtissues, and the continuous proliferation of SCs was observed.

#### Evaluation of neurotrophic factors secreted by nerve microtissues

**Fig. 3:**
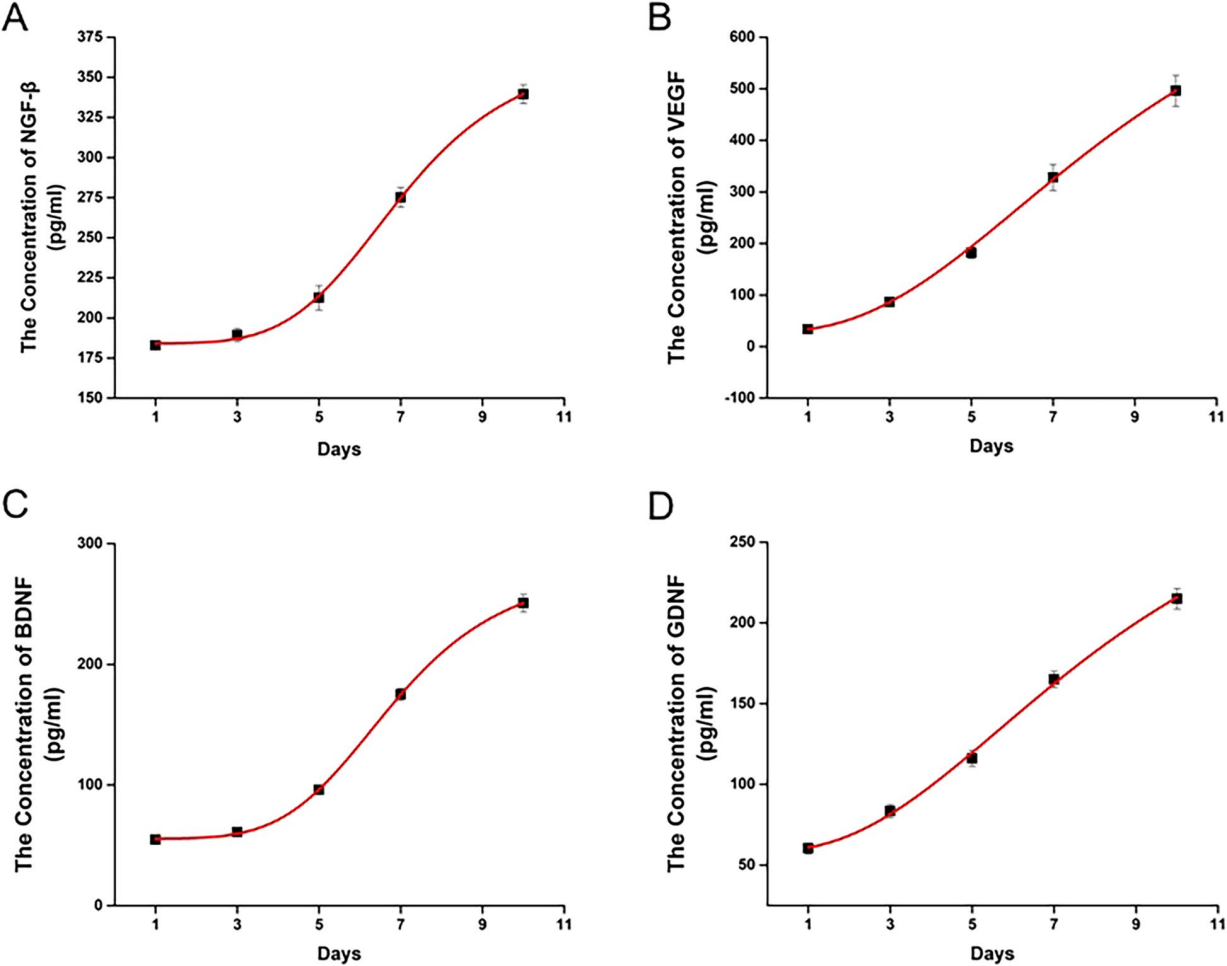
Evaluation of neurotrophic factors secreted by nerve microtissues. ELISA detected a rapid increase in the secretion of NGF-β, VEGF, BDNF and GDNF in living nerve microtissues after 3 days of culture. SCs Schwann cells, ELISA Enzyme linked immunosorbent assay

ELISA measurements showed that the concentrations of NGF-β, VEGF, BDNF, and GDNF in the culture medium increased rapidly after 3 days of nerve microtissue culture (Fig. 3).

#### SCs/DRG co-culture experiments

**Fig. 4:**
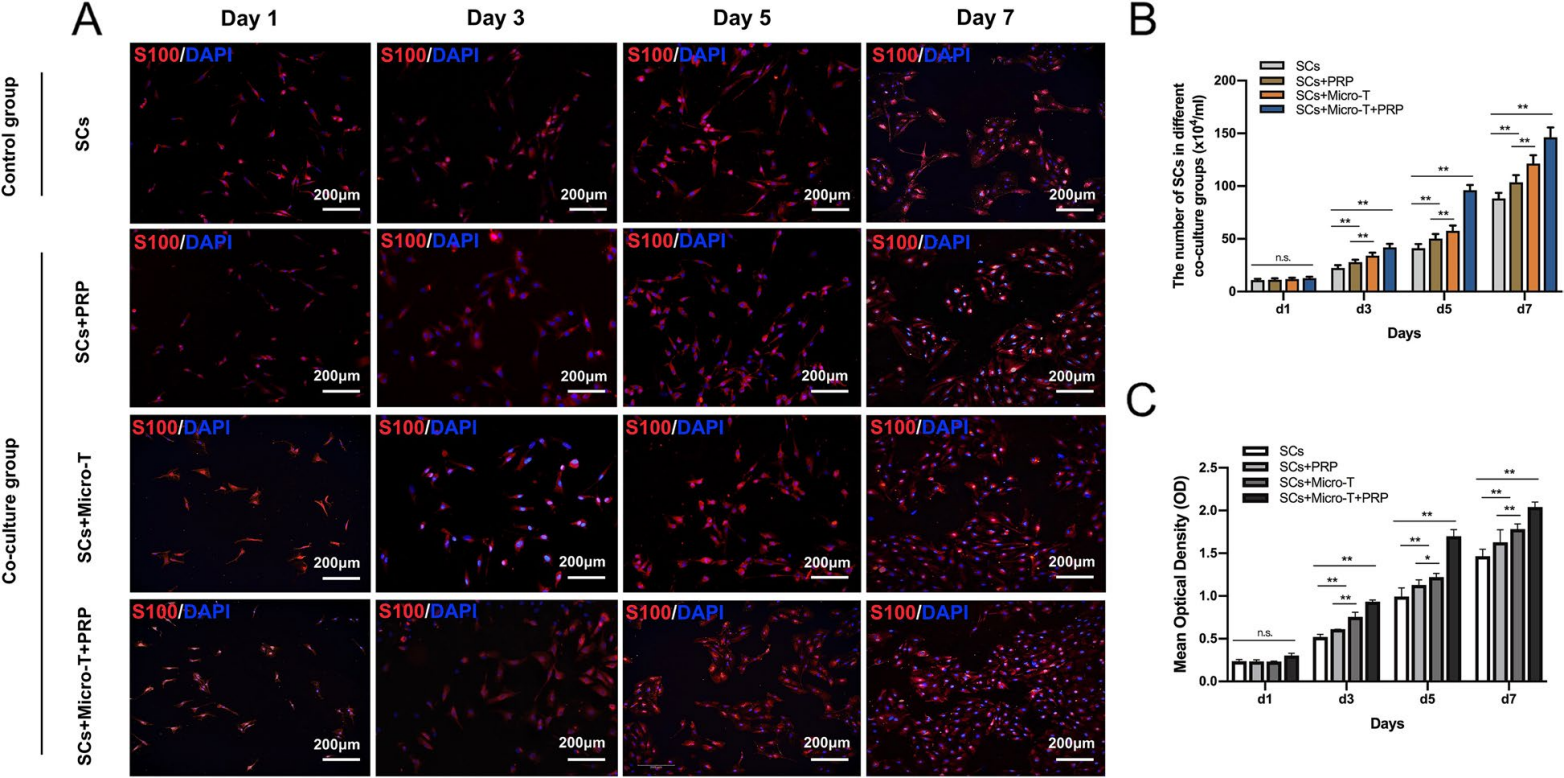
Proliferation evaluation of SCs co-cultured with living nerve microtissues or PRP supernatant. **A** SCs exposed to various groups during 1, 3, 5, 7 days of co-culture. SCs in the SCs + Micro-T + PRP group had faster proliferation, better spread and became more enlarged in shape than other groups from the third day of co-culture. The SCs count results were shown in (**B**). **C** CCK-8 colorimetric assay was performed to evaluate the effect of different groups on the proliferation of SCs (^*^P < 0.05, ^**^P < 0.01, n.s. *P* > 0.05). SCs Schwann cells, PRP platelet-rich plasma, Micro-T microtissues, CCK-8 Cell Counting Kit-8

As shown in Fig. 4A, SCs were detected by anti-S-100 immunofluorescence staining. When co-cultured with PRP supernatant or nerve microtissues, SCs proliferated faster than that of SCs cultured alone. In addition, SCs in the SCs + Micro-T + PRP group exhibited faster proliferation and better spread and became more enlarged in shape than other groups from the third day of co-culture. The SC count results are shown in Fig. 4B. After 3 days of co-culture, the CCK-8-based viability assays provided further evidence that the mean OD value of the SCs + Micro-T + PRP group was increased significantly compared with that of the other groups (*P* < 0.001) (Fig. 4C).

**Fig. 5:**
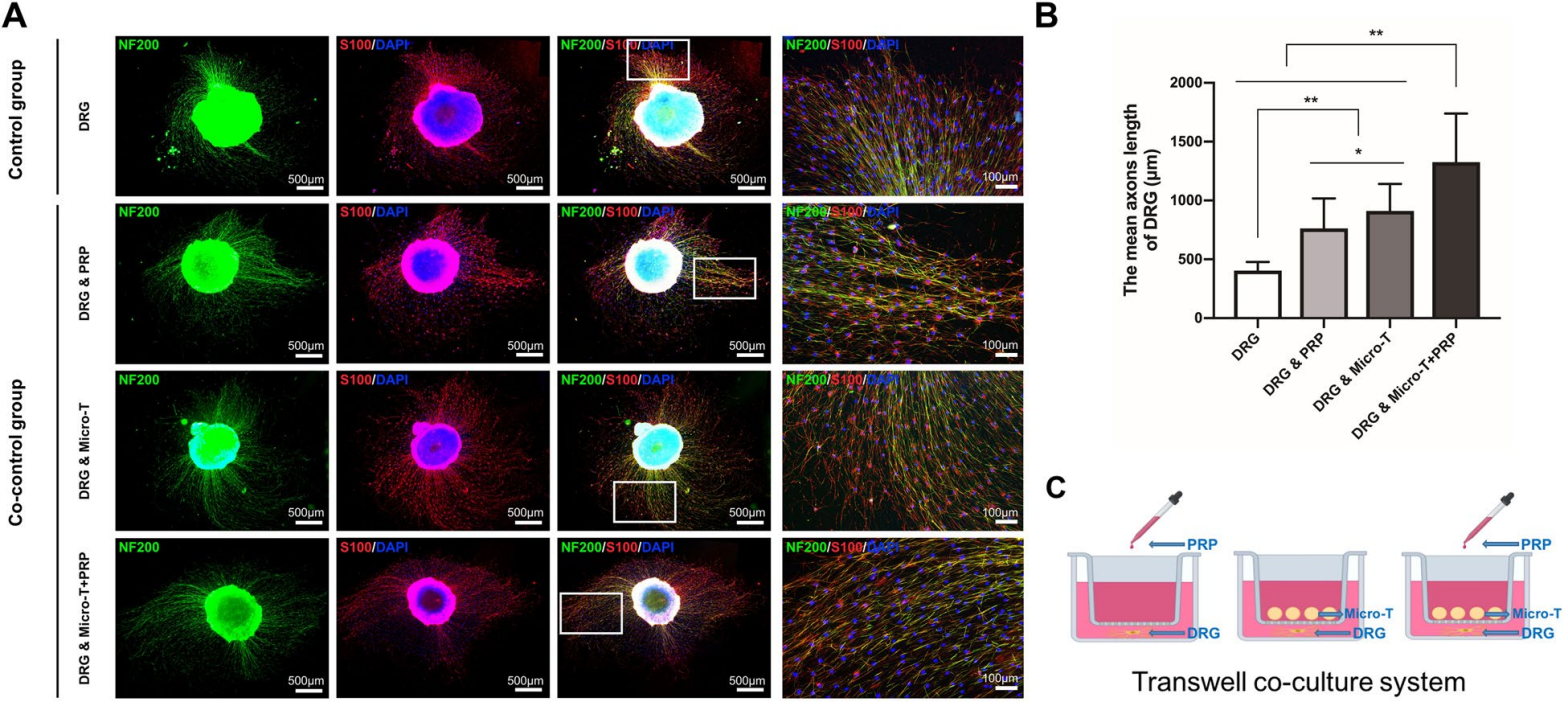
Proliferation evaluation of DRG co-cultured with living nerve microtissues or PRP supernatant. **A**-**B** The DRG in Micro-T + PRP group exhibited longer regenerated axons compared with those other co-culture groups and control group (^*^P < 0.05, ^**^P < 0.01). **C** Schematic of co-culture of DRG with nerve microtissue or PRP supernatant. DRG Dorsal root ganglions, Micro-T microtissues, PRP platelet-rich plasma

Similar results were obtained in the DRG co-culture experiment. The DRG in the Micro-T + PRP group exhibited longer regenerated axons than those in the other co-culture groups and the control group (all *P* < 0.001) (Fig. 5A-C).

#### Schwann cells migration study

**Fig. 6:**
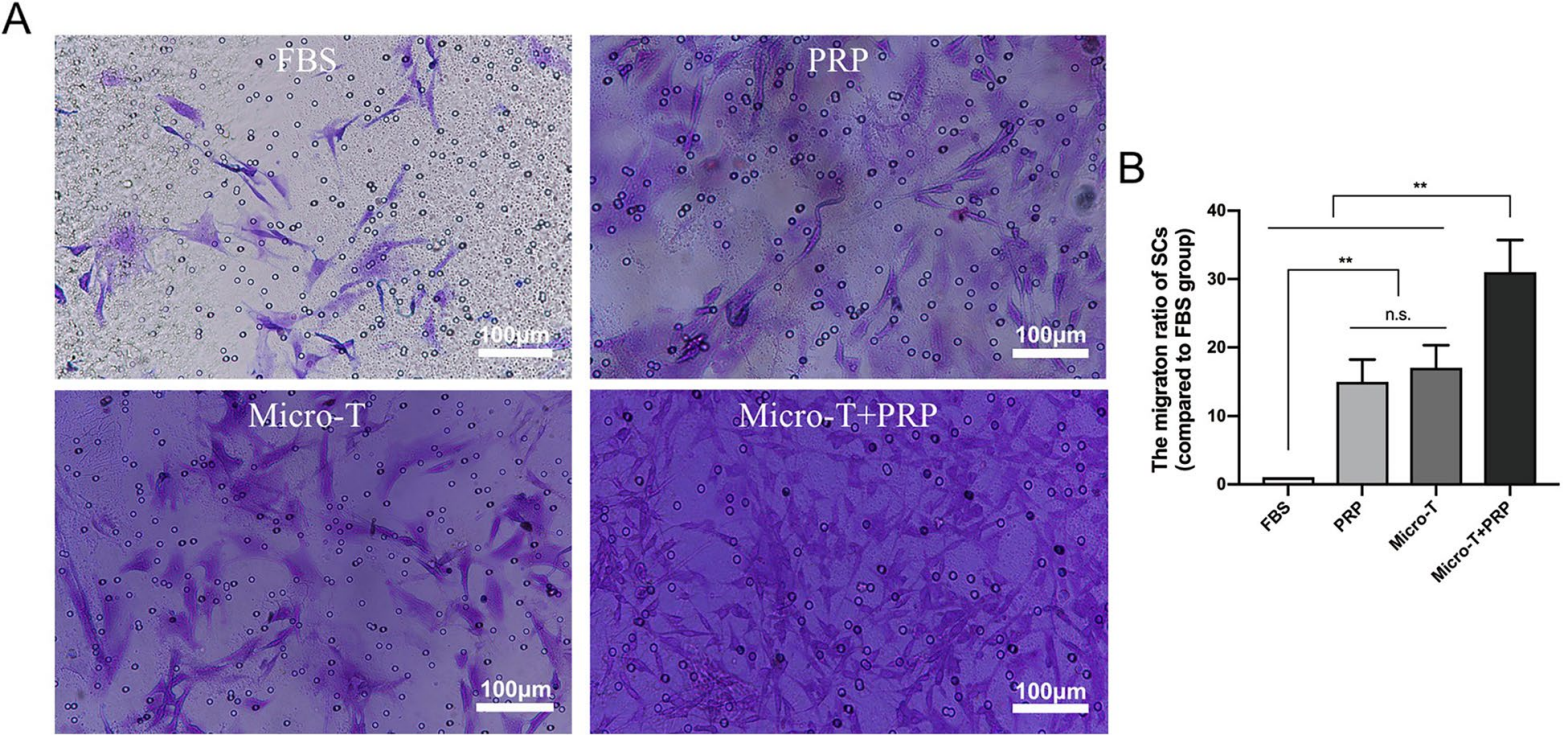
Schwann cells migration evaluation. **A**, **B** showed a significant increase in the migration with a combined application of the 5-day microtissue medium and PRP supernatant (^**^P < 0.01, n.s *P* > 0.05). FBS Fetal Bovine Serum, Micro-T microtissues, PRP platelet-rich plasma

As SCs motility and migration play pivotal roles in peripheral nerve regeneration, we examined whether nerve microtissues, PRP, and the combined application of the two affected the migration of SCs. The cells that migrated through the membrane were stained and counted (Fig. 6A, B). There was a significant increase in chemotactic potency with the combined application of 5-day microtissue medium and PRP supernatant. As shown in Fig. 6B, the highest migration ratio of SCs was evident after stimulation with the combination of microtissues and PRP.

## In vivo study

### Nerve recovery assessment

#### Nerve function evaluation

**Fig. 7:**
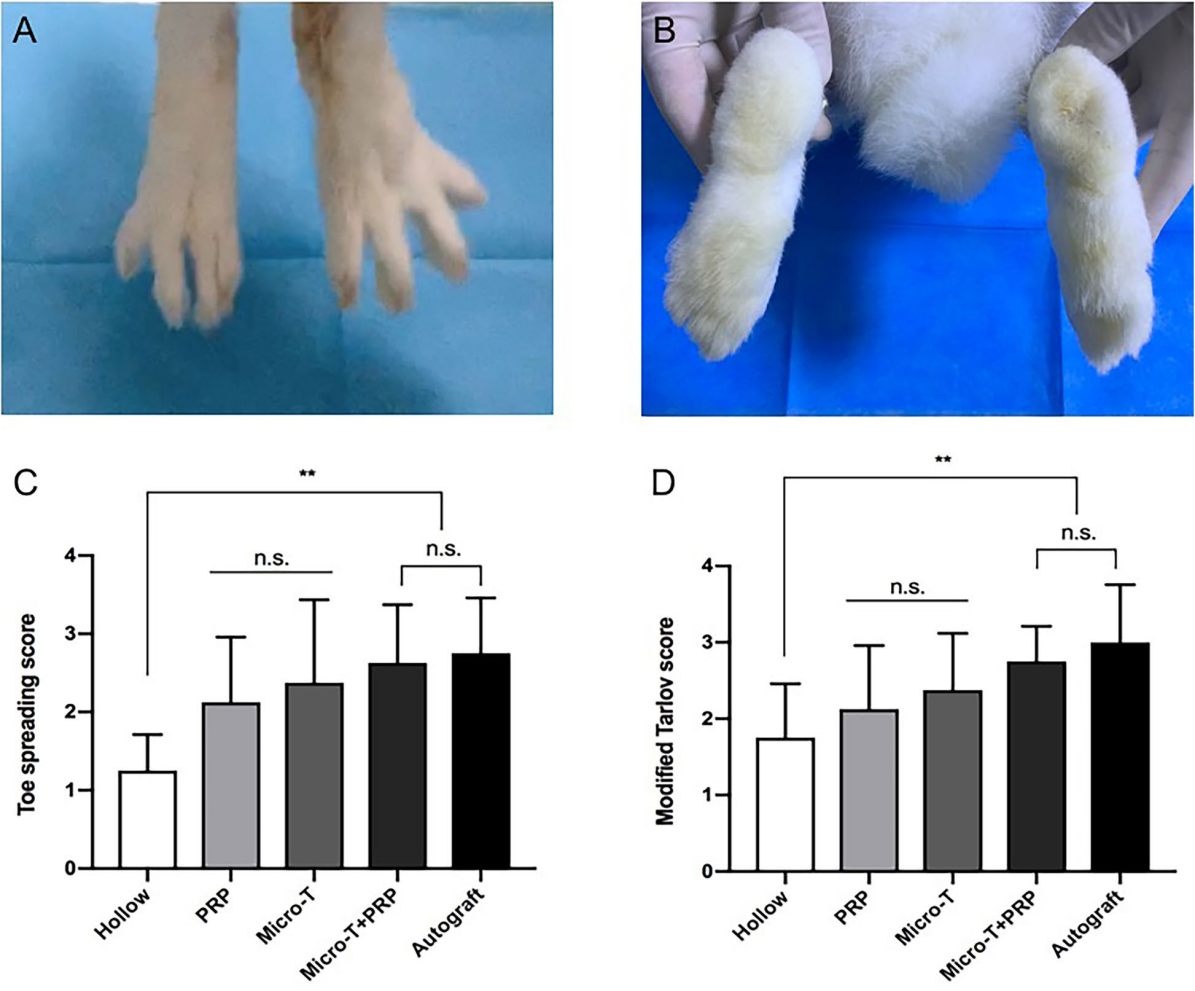
Nerve function evaluation after 12 weeks operation. **A**-**B** The foot ulcers were not obvious in the Micro-T + PRP group and Autograft group. **C** The toe spreading score evaluation among different groups. **D** The modified Tarlov score evaluation among different groups. ^**^P < 0.01, and n.s. not significant. Micro-T microtissues, PRP platelet-rich plasma

Nerve function was evaluated by the toe spreading score and the modified Tarlov score (*n* = 8 animals/group) (Fig. 7C, D). At 12 weeks after surgery, there was no significant difference in functional scores (FSs) between the Micro-T + PRP and Autograft groups (*P* > 0.05), but they were significantly higher than those of the Hollow group (*P* < 0.001). In addition, there were no significant foot ulcers in the Micro-T + PRP and Autograft groups (only one animal in each group) (Fig. 7A, B).

#### High-frequency ultrasound and CEUS evaluation of vein grafts

**Fig. 8:**
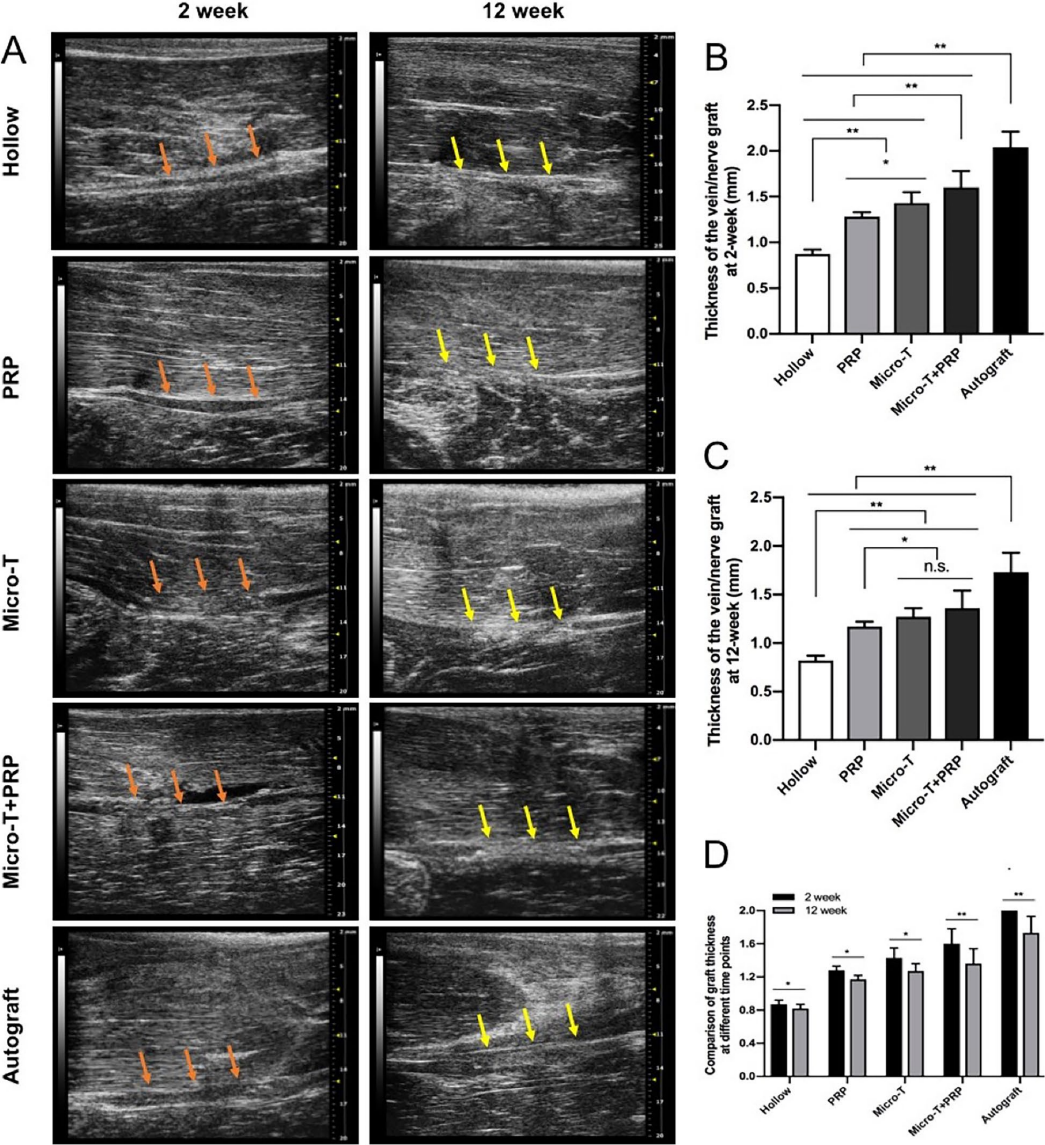
High-frequency nerve ultrasound examination was performed at 2 weeks and 12 weeks after operation. **A** High-frequency ultrasound images of the nerve graft on the surgical side. Statistical analyses were performed by calculating the thickness of the nerve graft at **B** 2 weeks and **C** 12 weeks. **D** Comparison of nerve graft thickness at different time points. ^**^P < 0.01, and n.s. not significant. Micro-T microtissues, PRP platelet-rich plasma

At 2 weeks after the operation, high-frequency ultrasound showed that the walls of the vein grafts in each group were clear and continuous and exhibited good continuity between the vein graft and nerve stumps (Fig. 8A). The vein grafts in all treatment groups were significantly thicker and had lower echogenicity than those in the Hollow group (Fig. 8A, B). At 12 weeks, significant venous collapse occurred in the Hollow group, while no venous collapse was observed in the other treatment groups. Meanwhile, the lumen of the vein was hyperechoic (Fig. 8A). The thickness of the vein grafts in the therapy groups was significantly reduced compared with that in the previous examination (Fig. 8A, C, D). In addition, the transplanted tibial nerve was thicker than the other vein grafts, but it also recovered significantly at 12 weeks.

**Fig. 9:**
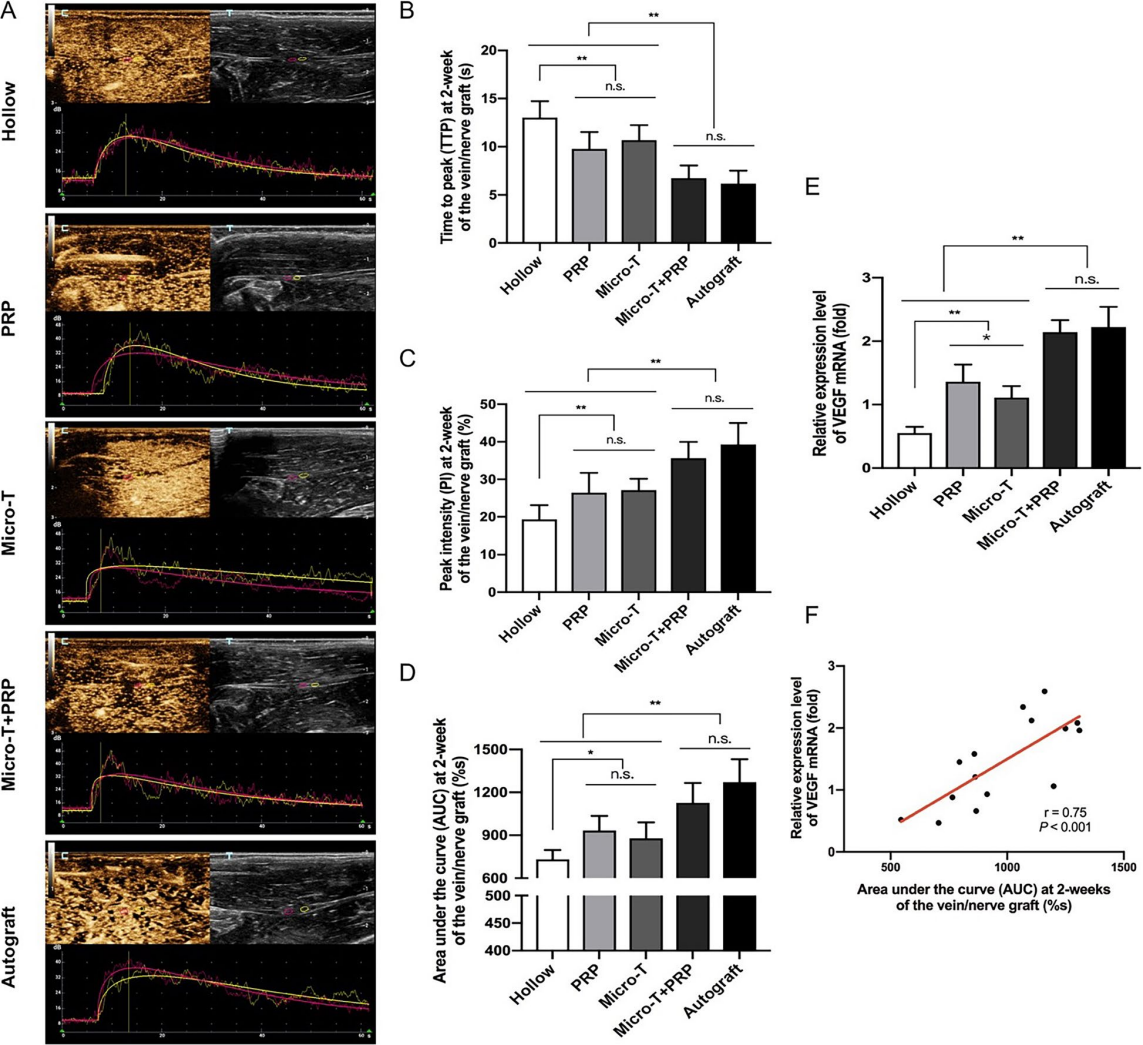
CEUS detection and VEGF mRNA analysis of nerve graft on the operated side at 12 weeks post-operation. **A** CEUS examination of regenerative vessels of nerve graft. The enhanced time was shown in the horizontal axis and the enhanced intensity was shown in the vertical axis. The time-intensity curves of CEUS image of nerve graft in the hollow group, PRP group, Micro-T group, Micro-T + PRP group and Autograft group were displayed. The pink and yellow ROIs represented the same two sampling frames, and they referred to repeated measurements at different parts of the nerve graft. Statistical analyses were performed by comparing the **B** TTP, **C** PI and **D** AUC on the CEUS in the nerve graft among groups. **E** Relative expression level analysis of VEGF mRNA. **F** Correlation analysis of the expression of VEGF mRNA and the AUC value. ^*^P < 0.05, ^**^P < 0.01, and n.s. not significant. CEUS contrast-enhanced ultrasonography, Micro-T microtissues, PRP platelet-rich plasma, ROI region of interest, TTP time to peak, PI peak intensity, AUC area under the curve

CEUS of the vein graft was quantitatively analyzed at 2 weeks after surgery (Fig. 9 A-F). The TTP, PI and AUC were obtained from a time-intensity curve. A higher PI and AUC and a lower TTP indicated better blood perfusion recovery. No significant difference in blood perfusion (comparison of TTP, PI, AUC) was observed between the Autograft and Micro-T + PRP groups (all *P* > 0.05), and both groups were superior to the other three groups (all *P* < 0.01) (Fig. 9B-D). Although the blood perfusion of the PRP and Micro-T groups was significantly lower than that of the combined application, it was still significantly higher than that of the Hollow group (Fig. 9B-D).

#### Quantitative real-time RT-PCR (qRT-PCR)

Following 2 weeks of treatment, VEGF mRNA expression was significantly upregulated in the Micro-T + PRP and Autograft groups compared with the other groups (*n* = 3 animals/group) (*P* < 0.01). Meanwhile, the expression of VEGF was had a positive linear correlation with the AUC value, representing microvascular perfusion on CEUS (*r* = 0.75, *P* < 0.001) (Fig. 9F).

#### Macroscopic evaluation of vein graft adherence

**Fig. 10:**
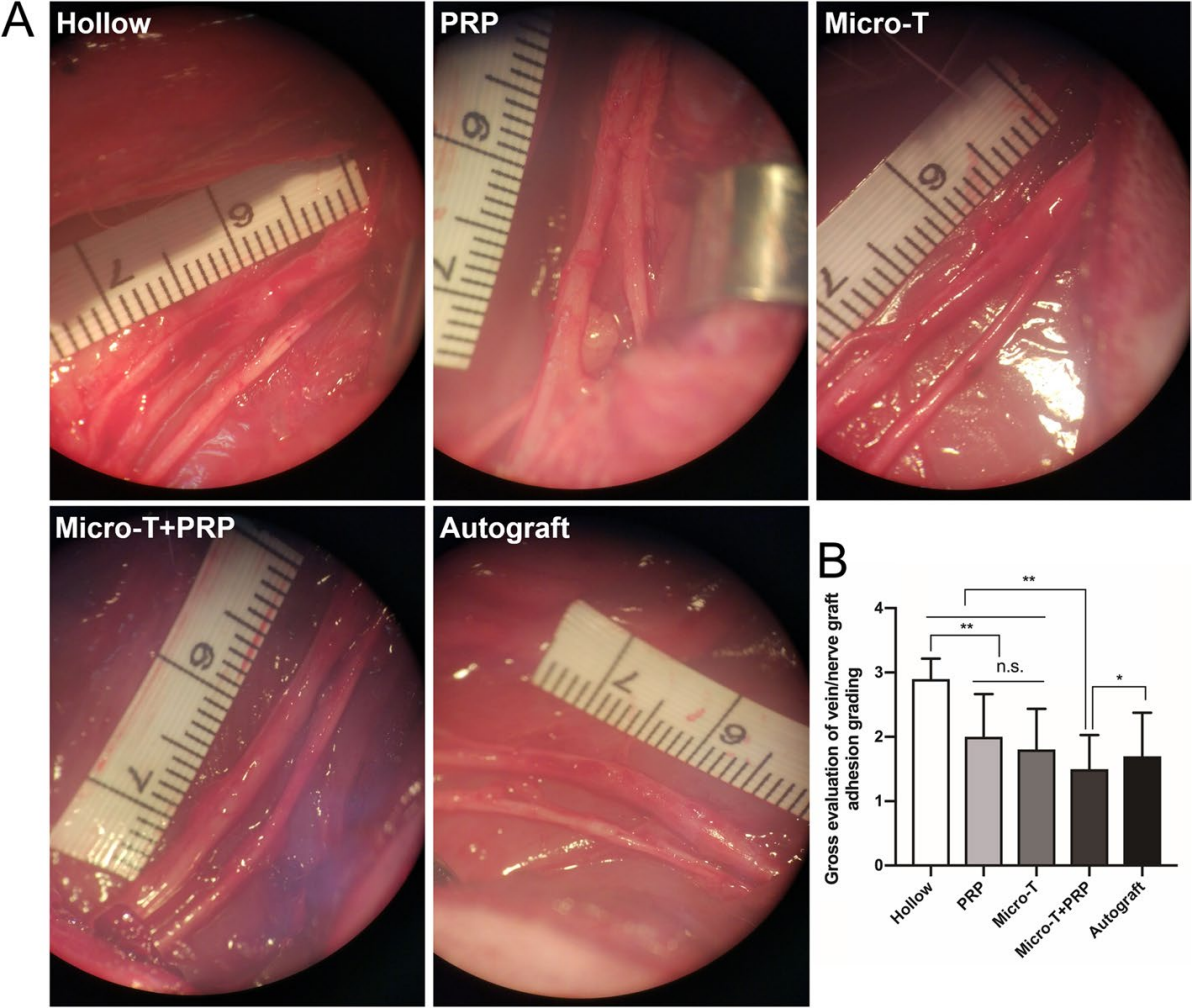
The degree of nerve graft adherence assessment. **A** A representative gross view of nerve grafts in each group after blunt dissection. **B** Statistical analyses were performed by comparing the degree of nerve graft adherence between each group. Grade I no dissection or mild blunt dissection; Grade II some vigorous blunt dissection required; Grade III sharp dissection required. ^*^P < 0.05, ^**^P < 0.01, and n.s. not significant. Micro-T microtissues, PRP platelet-rich plasma

No graft fracture was observed at 12 weeks after surgery. The vein grafts treated with saline (Hollow group) demonstrated dense scar tissue formation surrounding the repair site, which was difficult to dissect from the surrounding tissue, and part of the venous wall collapsed. However, thin, lucent membrane-like tissue surrounding the repair site in the Micro-T + PRP group had the least adhesion degree and required only mild blunt dissection. The perineural adhesion in the PRP and Micro-T groups was significantly lower than that in the Hollow group (all *P* < 0.01), and there was no significant difference between the two groups (*P* > 0.05) (Fig. 10A, B).

#### Electrophysiological recovery evaluation

**Fig. 11:**
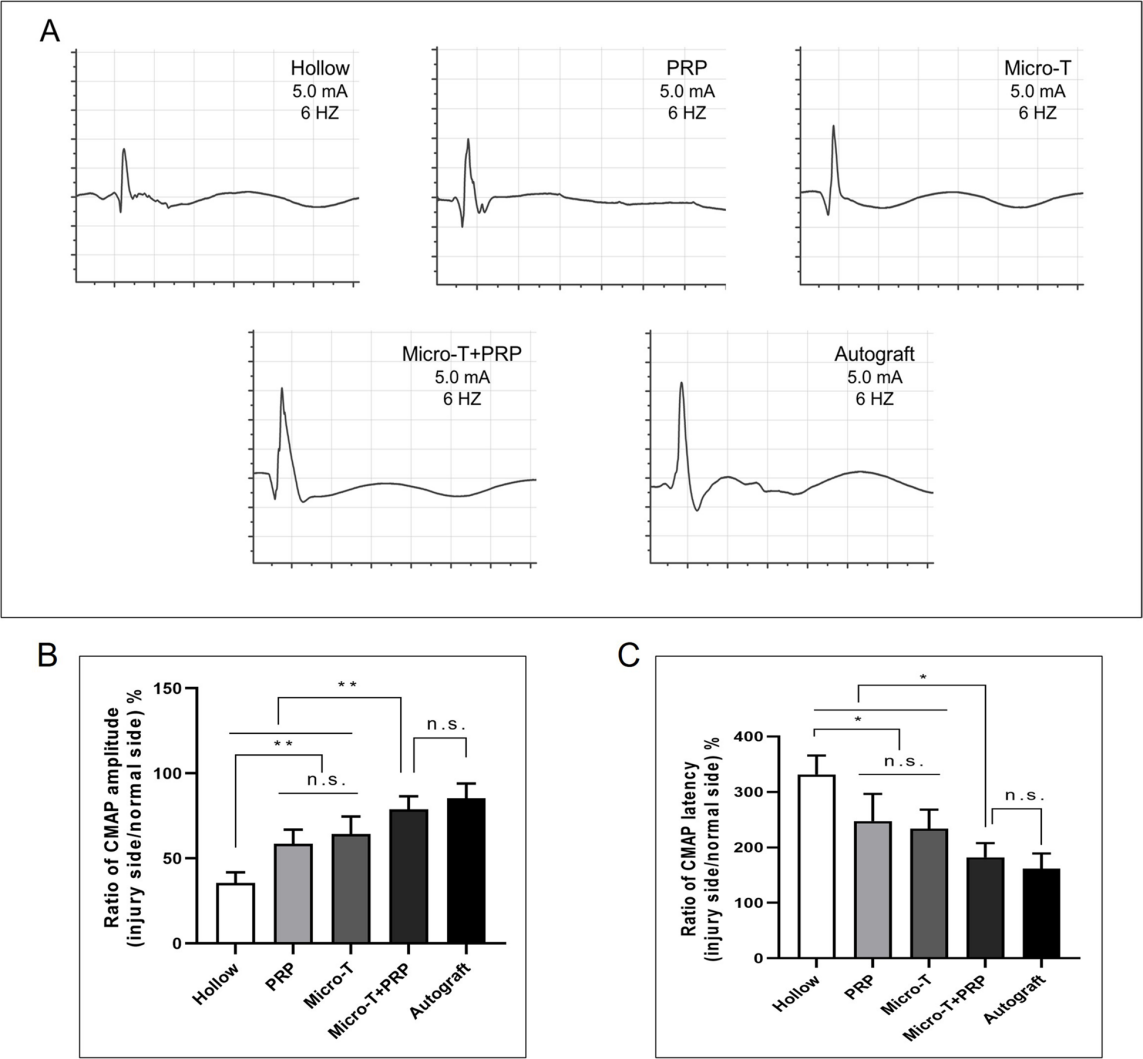
The electrophysiological recovery assessment at 12 week. **A** Representative CMAP recordings from the operated side of each group. **B**-**C** Tibial nerve CMAP amplitude and CMAP latency comparisons among groups. ^*^P < 0.05, ^**^P < 0.01, and n.s. not significant. Micro-T microtissues, PRP platelet-rich plasma

At 12 weeks after the surgery, the ratio of CMAP amplitude and the ratio of CMAP latency were significantly improved in the Micro-T + PRP group compared with the PRP group, Micro-T and Hollow groups (all *P* < 0.05), and were similar to those in the Autograft group (*P* > 0.05) (Fig. 11A-C). Additionally, no significant difference was shown in the electrophysiological results between the Micro-T and PRP groups (Fig. 11A-C).

#### Histological evaluation of regenerated nerves at an early stage

**Fig. 12:**
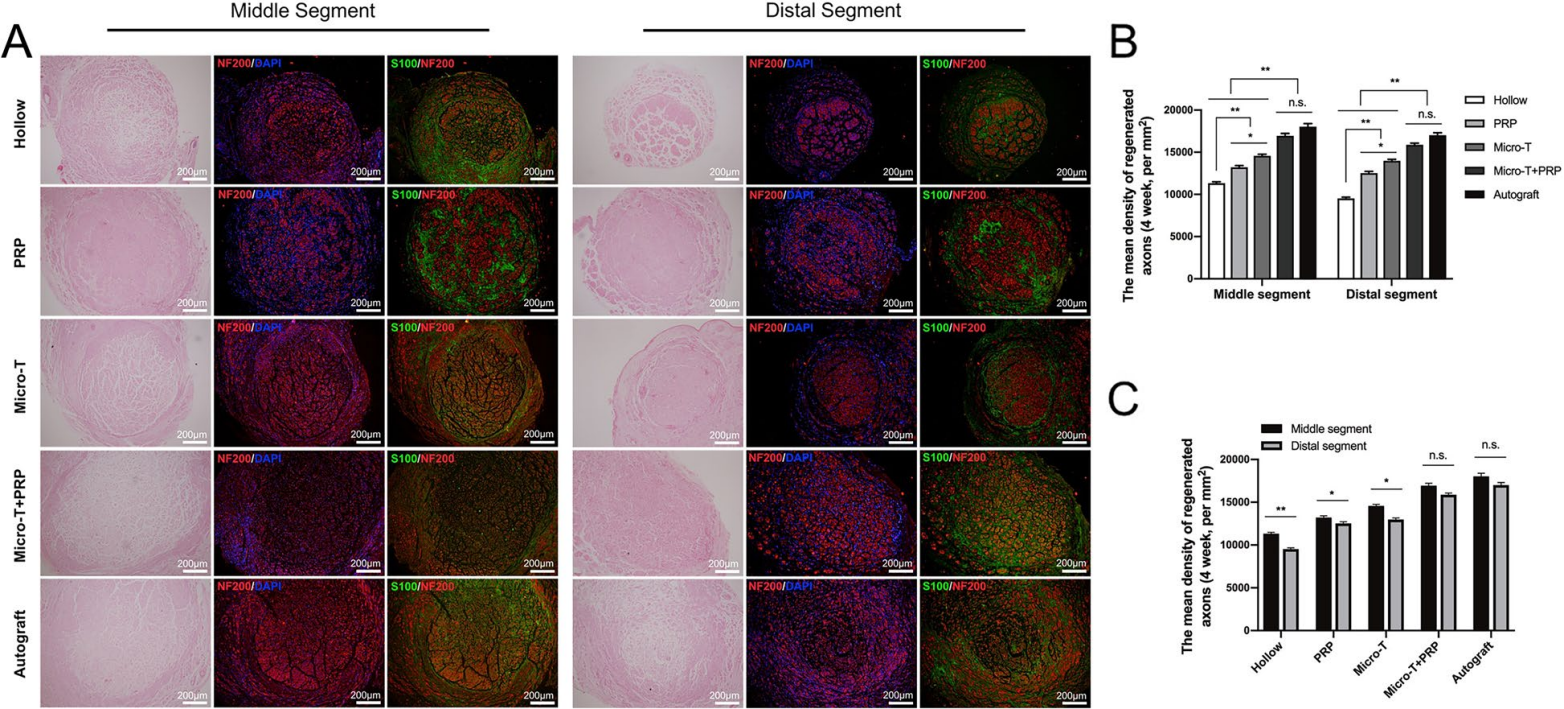
Histological evaluation of regenerated nerves at 4 weeks. **A** H&E staining and immunofluorescence staining were performed at middle and distal segment separately. Anti-Neurofilament 200 was used to stain regenerative axons (red) and anti-S100 antibody was used to stain regenerative myelin sheath (green). Nuclei were stained with DAPI (blue). Statistical analyses were performed by calculating **B**-**C** the mean density of regenerative axons between groups. ^*^P < 0.05, ^**^P < 0.01, and n.s. not significant. Micro-T microtissues, PRP platelet-rich plasma

At 4 weeks, in the histological analyses of the middle and distal segments of the grafts, for all groups, regenerating axons grew from the proximal to the distal end of the vein lumen (Fig. 12A). The density of regenerated axons in the middle segment in each group was compared, as shown in Fig. 12B. Except for the Autograft group, the density of regenerated axons was the highest in the Micro-T + PRP group and the lowest in the Hollow group; however, no significant difference was observed between the Micro-T + PRP and Autograft groups (Fig. 12B).

#### Morphometrical assessment of regenerative nerves

**Fig. 13:**
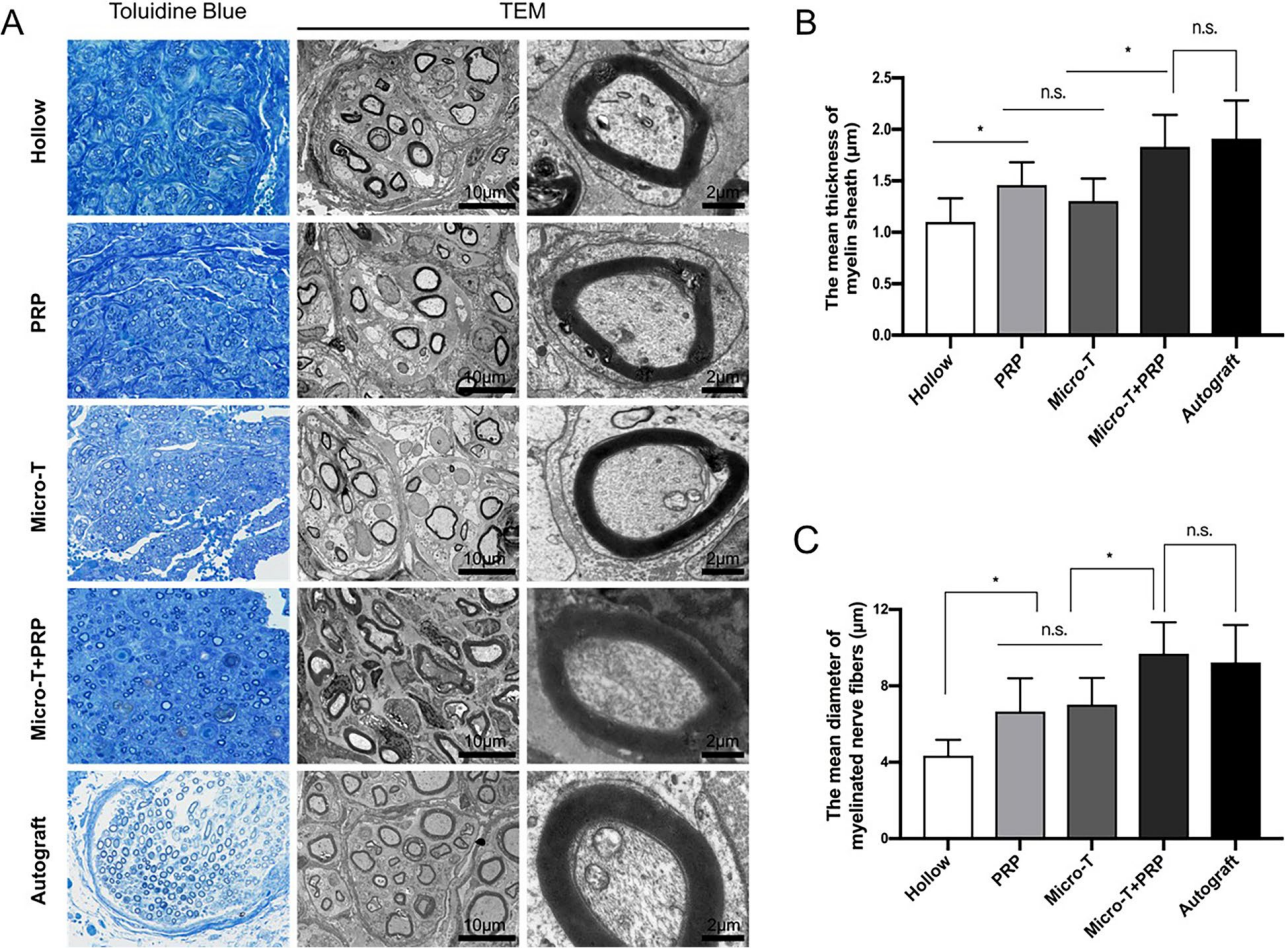
Transverse semithin sections and transmission electron micrography evaluation of regenerated myelinated nerve fibers in the distal portion at 12 weeks after operation. **A** A representative image of regenerated myelin sheath in each group. Histomorphometric analysis was performed by calculating **B** the mean thickness of myelin sheath and **C** the mean diameter of the myelinated nerve fibers. ^*^P < 0.05, ^**^P < 0.01, and n.s. not significant. Micro-T microtissues, PRP platelet-rich plasma

The morphometrical results for the distal region of graft sites at 12 weeks are shown in Fig. 13 A-C. At the light and electron microscopy levels, there were clear differences in axonal and myelin morphology between the microtissue+PRP- supplemented group and Micro-T/PRP/saline-filled veins. The Micro-T + PRP group exhibited significantly higher myelin sheath diameter and thickness than the PRP, Micro-T, and Hollow groups (all *P* < 0.01), which was similar to the results in the Autograft group (*P* > 0.05).

### Triceps surae muscle recovery assessment

#### Multimodal ultrasound evaluation

##### High-frequency ultrasound evaluation

**Fig. 14:**
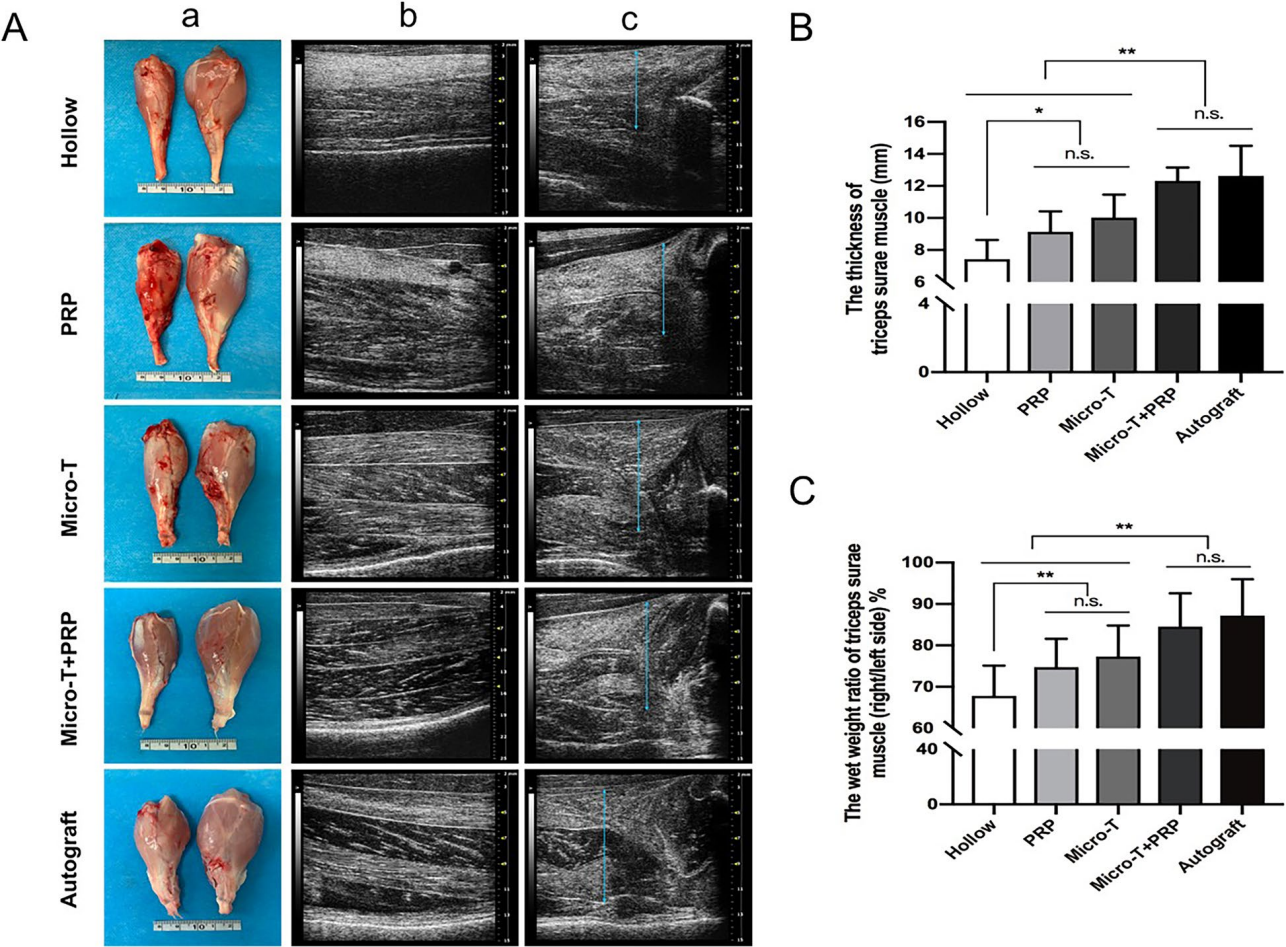
Macroscopic evaluation and high-frequency ultrasound evaluation of the targeted muscle. (**A**) Images of the targeted muscle. (a) The gross image; (b) The triceps surae muscle in the longitudinal section; (c) The thickness of the triceps surae muscle in the transverse scan. (B) Comparison of the thickness of triceps surae muscle in each group. (C) Analysis of the wet weight ratio of triceps surae muscle between groups. ^*^P < 0.05, ^**^P < 0.01, and n.s. not significant. Micro-T microtissues, PRP platelet-rich plasma

Twelve weeks after surgery, the target muscle of all the repaire groups showed atrophy to varying degrees, which manifested as decreased muscle thickness, increased muscle echogenicity, and blurred muscle texture characteristics. The Micro-T + PRP and Autograft groups had the least targeted muscle atrophy and had the best muscle thickness, which was consistent with the gross anatomy and wet weight ratio of the muscle (Fig. 14A, B, C).

##### SWE and AngioPLUS imaging evaluation

**Fig. 15:**
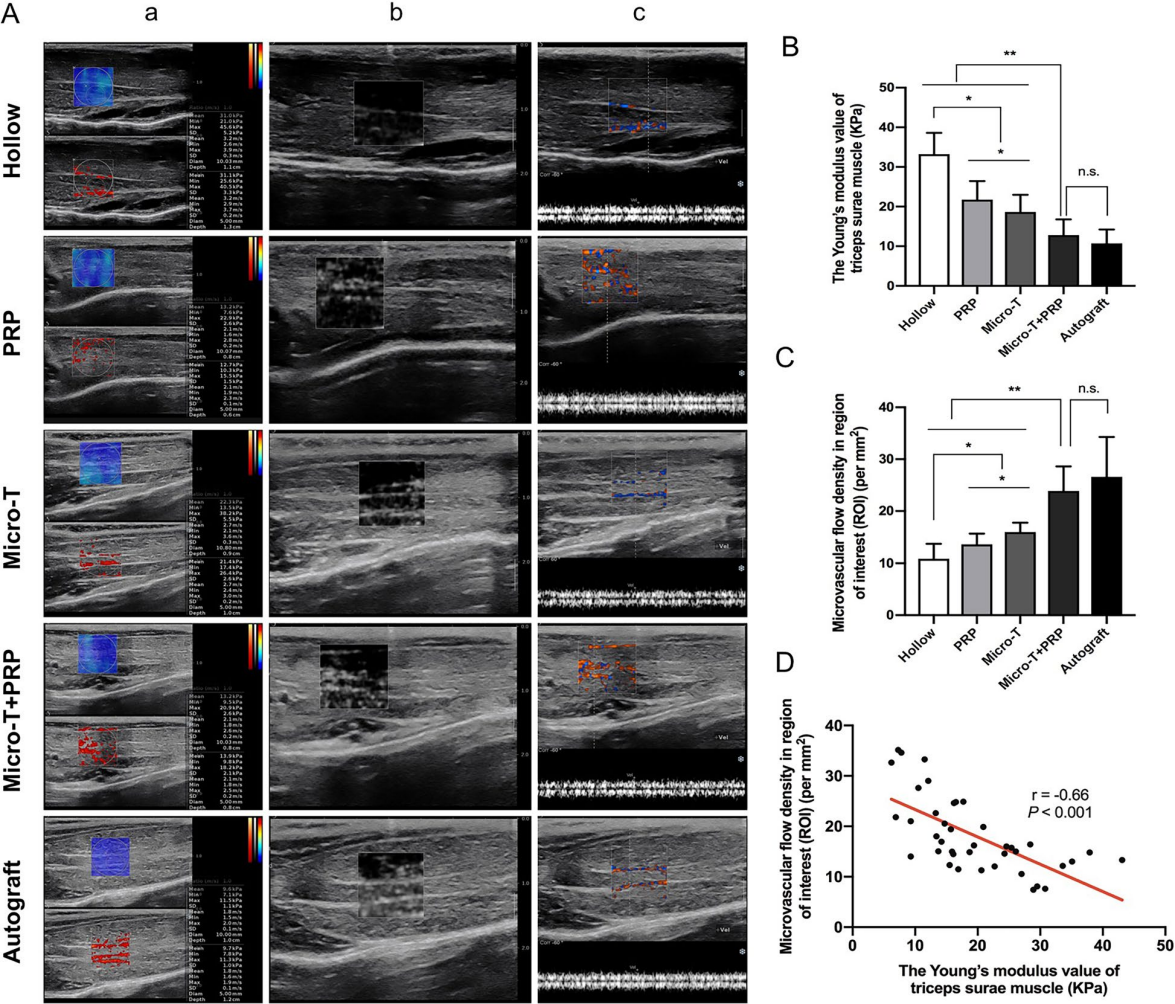
Shear wave elastography (SWE) and Angio PlaneWave UltrasenSitive (AngioPLUS) imaging evaluation. (A) SWE and AngioPLUS images were diaplayed simultaneously on the screen (a), and the monochrome pattern of the microvascular muscle was also displayed (b); (c) Spectral Doppler ultrasonography confirmed that the image shown by Angioplus was microvessel in the triceps surae muscle. Statistical analyses were performed by calculating (B) the Young’s modulus value of triceps surae muscle, and (C) the microvascular flow density in region of interest. ^*^P < 0.05, ^**^P < 0.01, and n.s. not significant. Micro-T microtissues, PRP platelet-rich plasma

Simultaneous SWE and AngioPLUS examinations showed the relationship between the muscle stiffness and the microvascular flow density of targeted muscle after nerve repair in eight animals in each group. The evaluation results of the Micro-T + PRP group were as good as those of the autologous nerve transplantation group; however, compared with the Hollow, PRP and Micro-T groups, the targeted muscle in the Micro-T + PRP group had the lowest Young’s modulus value (all *P* < 0.001), while the corresponding microvascular flow density was the highest (all *P* < 0.001) (Fig. 15A-C). In addition, through correlation analysis, a linear negative correlation between the stiffness and the microvascular flow density was observed in the reinnervated triceps surae muscle (*r* = − 0.66, *P* < 0.001) (Fig. 15D).

##### CEUS evaluation

**Fig. 16:**
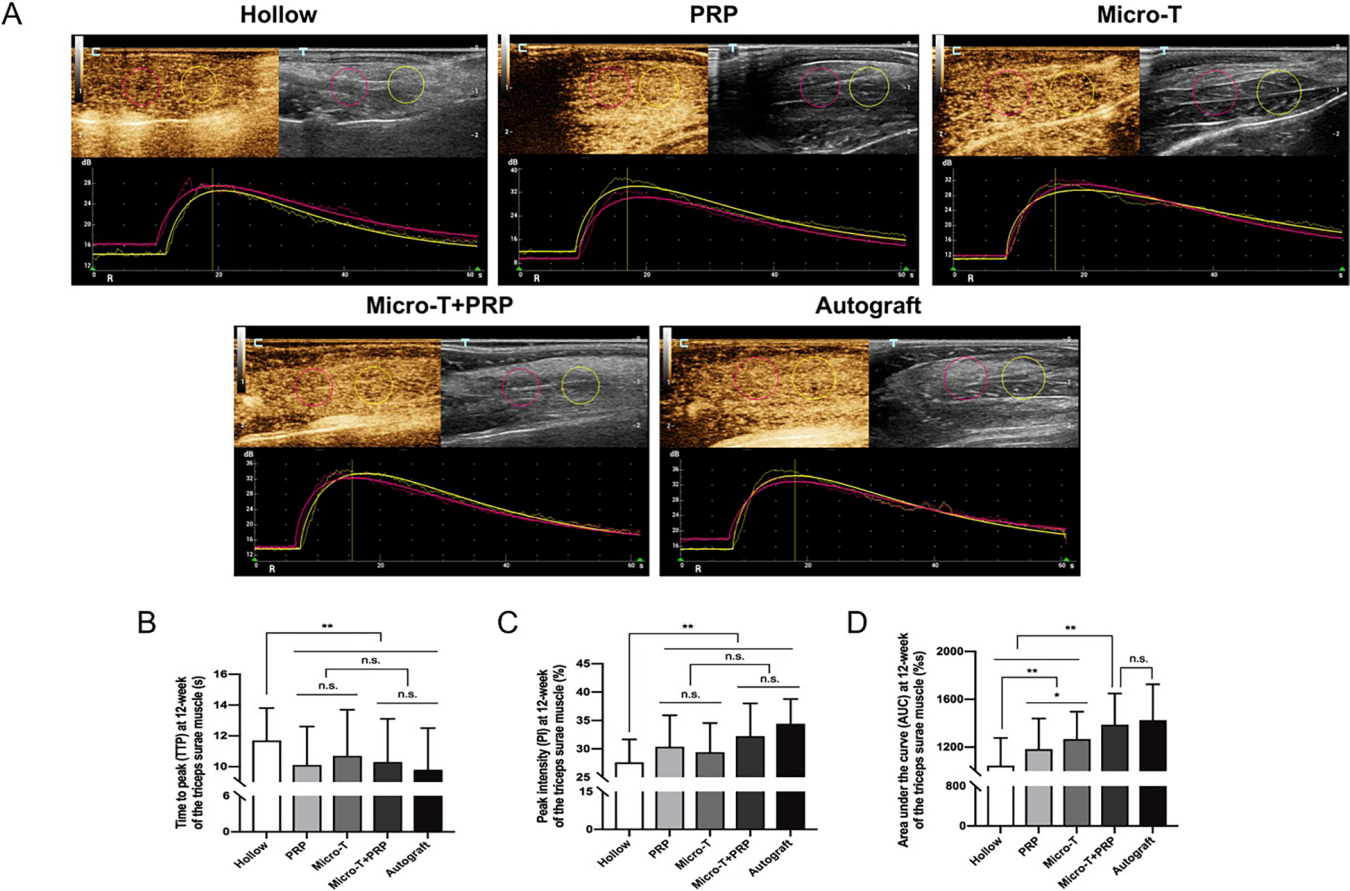
CEUS detection of targeted muscle at 12 weeks. **A** CEUS examination of triceps surae muscle. **A** Time-intensity curves of the triceps surae muscle (proximal: pink; distal: yellow) obtained by CEUS. The vertical axis represented the intensity and the horizontal axis displayed the time after injection of SonoVue. Statistical analyses were performed by comparing the **B** TTP, **C** PI and **D** AUC on the CEUS in the triceps surae muscle among groups. ^*^P < 0.05, ^**^P < 0.01, and n.s. not significant. CEUS contrast-enhanced ultrasonography, Micro-T microtissues, PRP platelet-rich plasma, TTP time to peak, PI peak intensity, AUC area under the curve

According to the time-intensity curve analysis in five animals in each group, the AUC value in targeted muscles of the Micro-T + PRP group were significantly higher than those of the Hollow, PRP, and Micro-T groups at 12 weeks after the operation (all *P* < 0.001) (Fig. 16A, D). In addition, there was no significant difference in muscle blood perfusion parameters, such as TTP, PI and AUC, between the Micro-T + PRP and Autograft groups (all *P* > 0.05) (Fig. 16B-D).

### Morphological evaluation

**Fig. 17:**
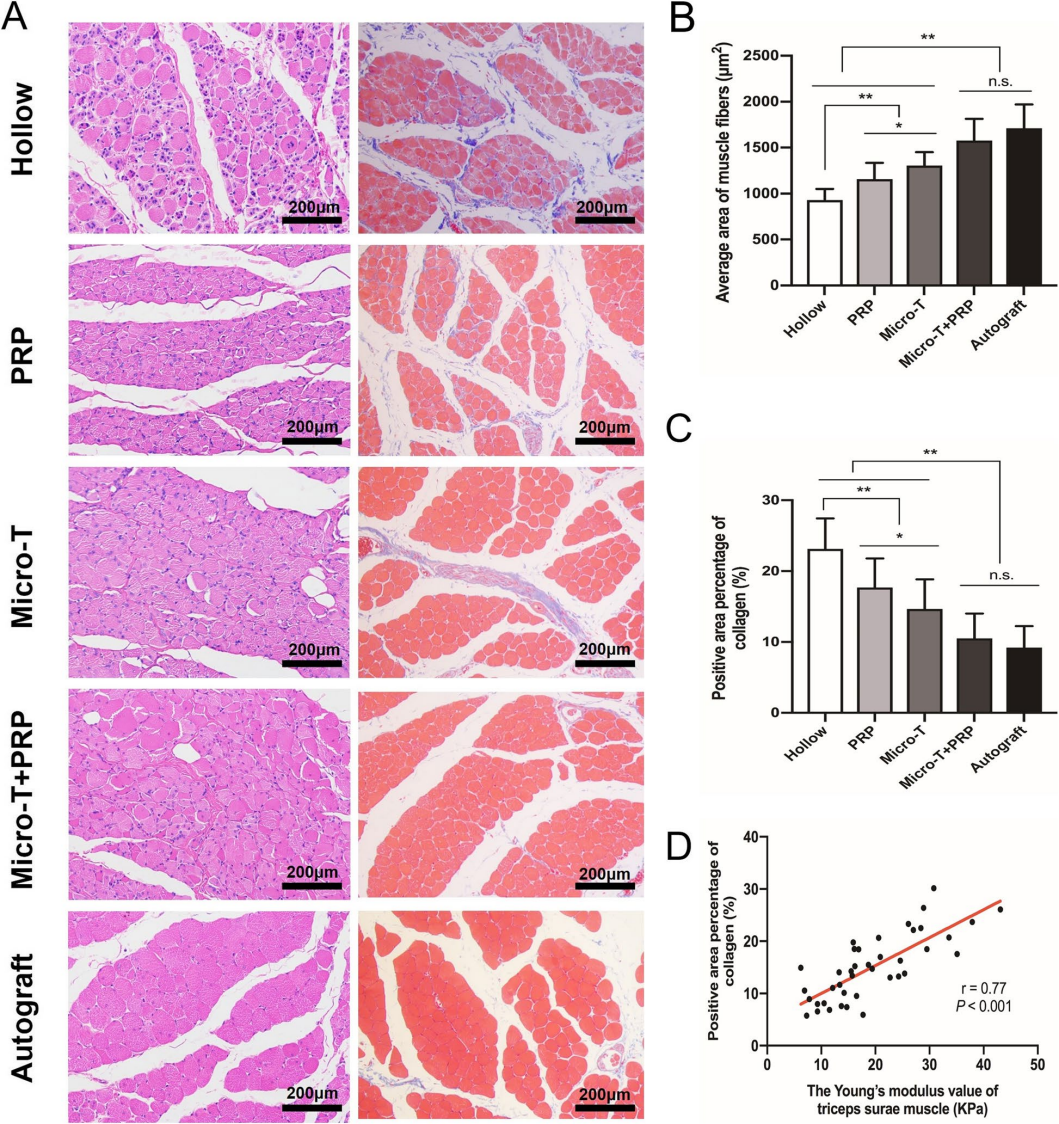
Histopathological evaluation of the targeted muscle. **A** Two types of image were shown for each group: H&E stained images and Masson’s trichrome-stained images. **B** Statistical analyses of average cross-sectional area of muscle fibers. **C** Statistical analyses of the mean percentage of collagen area of the muscle. **D** Correlation analysis of the positive area percentage of collagen and the Young’s modulus value of the triceps surae muscle. ^*^P < 0.05, ^**^P < 0.01, and n.s. not significant. Micro-T microtissues, PRP platelet-rich plasma

In the histological observations, the Hollow group presented significant invasion of connective tissue, centralized nuclei, and small fibre diameters, which were characteristics of denervated atrophic muscle fibres. The Micro-T + PRP and Autograft groups presented histomorphological similarity to each other, which was superior to the other groups. Great histological improvement was shown in the PRP and Micro-T groups compared with the Hollow group, although they still exhibited some centralized nuclei and slight fascicular disorganization (Fig. 17A).

On the other hand, in the triceps surae muscle, it was observed that the Autograft group had the highest mean cross-sectional area of muscle fibres, and the lowest mean value was observed in the Hollow group. The Micro-T + PRP and Autograft groups had the smallest percentage of collagen-positive area among the experimental groups (*P* < 0.001) (Fig. 17B, C). The collagen area was positively correlated with the elastic modulus(*r* = 0.77, *P* < 0.001) (Fig. 17D).

## Discussion

After traumatic peripheral nerve injury, timely and effective treatment and objective and reliable postoperative evaluation are necessary to promote the functional recovery of these patients. The results of this study showed that the mixture of nerve microtissues isolated from small pieces of predegenerated tibial nerves and autologous PRP transplanted within an autologous vein guide could enhance peripheral nerve regeneration across a gap of 12-mm in rabbits. Multimodal ultrasound examination, a simple and non-invasive evaluation method, has a significant correlation with histopathological results and can provide a reference for prognosis.

After severe nerve injury, substance loss of substance results in a significant gap between the nerve stumps that prevents repair by direct epineural suture. In addition to autologous nerve transplantation, tubulization is an alternative repair method to offer mechanical guidance as well as an optimal environment for the advancing axonal sprouts. Ideal tubulization materials should be biocompatible, bioresorbable, permeable, flexible, biodegradable, nontoxic, and nonimmunogenic [25]. Moreover, a transparent conduit is preferred so that the nerve can be observed while being introduced into it [26]. Hence, blood vessels can be used as tubular scaffolds, and autologous vein grafts are usually preferred over artery grafts in bridging nerve defects due to their minimal morbidities and ability to secrete NGF [27].

**Fig. 18:**
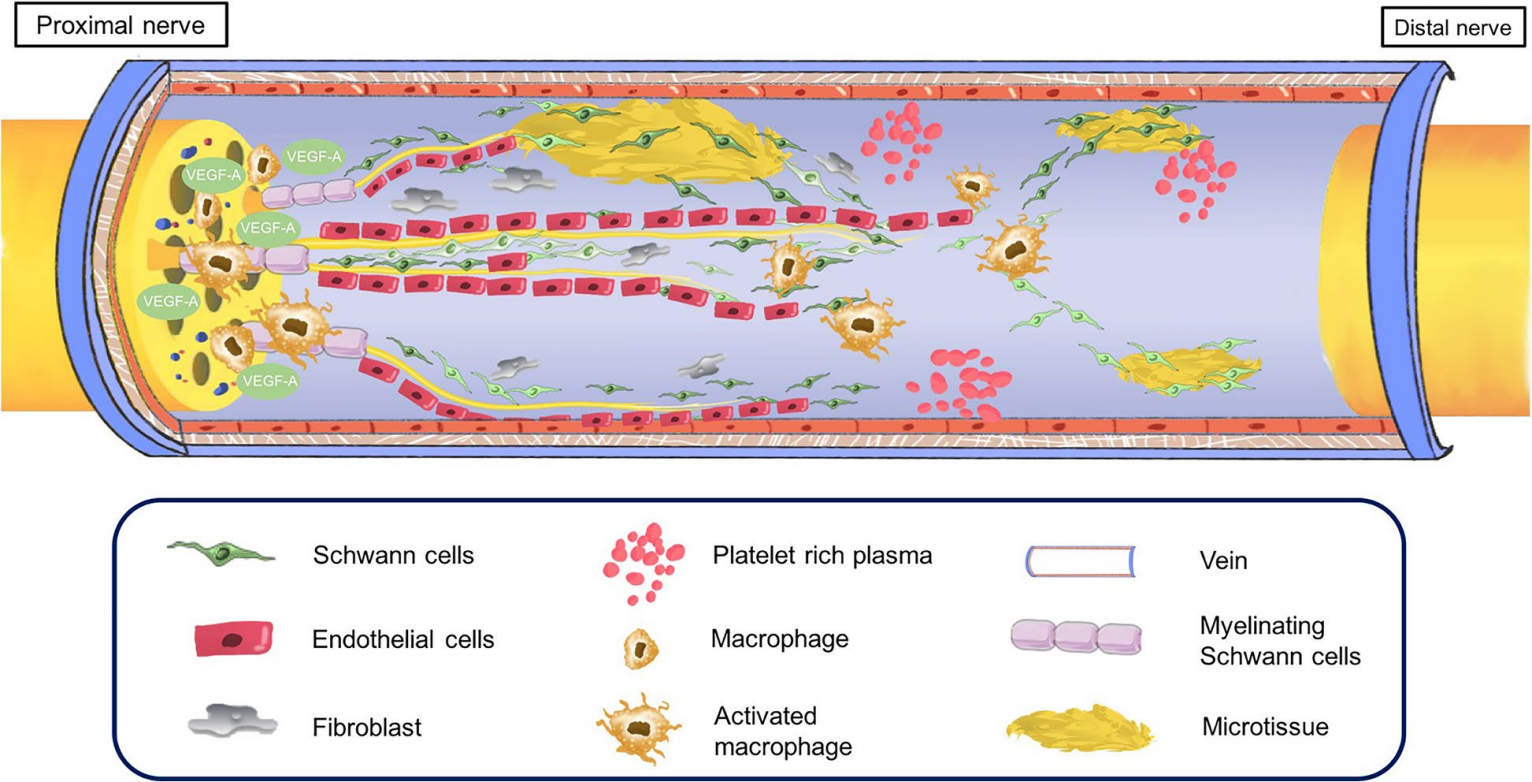
Schematic diagram of peripheral nerve defect repair using autogenous vein grafts filled with PRP and active nerve microtissues. After nerve injury, SCs recruit macrophages to facilitate the removal of myelin axonal remnants. At the same time, hypoxia within the bridge is selectively sensed by macrophages, which secrete VEGF-A to induce the formation of polarized vasculature in the bridge area. Nerve microtissues could be decomposed into a large number of SCs, which could secrete a large quantity of neurotrophic factors to promote the generation of blood vessels and the proliferation and migration of SCs. However, SCs showed a limited proliferative capacity. The addition of PRP may remedy this limitation. PRP, which contains various growth factors that are associated with peripheral nerve regeneration, can further stimulate SCs play the above roles in different ways. Thus promoting axon extension along the Büngner brand, and the SCs complete the nerve regeneration process by remyelinating the newly formed axons

A potential problem associated with the use of veins is the possibility of vein wall collapse due to tissue compression. In our study, filling the vein lumen with nerve microtissues or activated PRP gel enhanced the resistance of the vein. Another disadvantage of vein grafts is the presence of valves inside the lumen. Ahmed [28] et al. compared standard vein grafts with inverted vein grafts for nerve repair, and there was no significant difference elicited in the histological, morphometric or muscle mass measurements between groups. Our study also showed that the valves did not induce lumen obstruction in the reversed vein, and regenerating axons could grow from the proximal to the distal end of the vein lumen based on NF200 staining. Another more critical problem was the lack of SCs in the transplanted vein lumen, which was one of the key players in the repair of the peripheral nervous system and a limiting factor for the regeneration of long-distance gaps [29]. After nerve injury, SCs recruit macrophages to facilitate the removal of myelin axonal remnants [30]. Additionally, they proliferate and align to form the Büngner band, which acts as the guide structure for the regenerating axon [31]. After the axon elongates along the Büngner brand, the SCs complete the nerve regeneration process by remyelinating the newly formed axons [32]. However, SCs showed a limited proliferative capacity. A reason for the unsatisfactory regeneration of axons across wide gaps was that SCs had a reduced capacity to form a full-length Büngner band and, therefore, failed to guide the outgrowing axon [29]. Several studies have shown that the decreased capacity of SCs to support axonal regeneration is caused by SCs atrophy through long-term denervation and downregulation of neurotrophic factors such as neuromodulin-1 [33]. In this study, in vitro experiments showed that nerve microtissues could be decomposed into a large number of SCs, which could secrete a large quantity of neurotrophic factors to promote the proliferation and migration of SCs and axon regeneration of the DRG (Fig. 18). Therefore, we transplanted autologous nerve microtissues into venous catheters and found that compared with the Hollow group, axon regeneration, target muscle atrophy and limb functional recovery were significantly improved.

It is worth noting that autologous SCs transplantation has the following advantages in the process of axonal regeneration. First, SCs are the main source of neurotrophic factors [34]. SCs not only increase cell survival and promote the regeneration of blood vessels by secreting a variety of neurotrophic factors such as nerve growth factor (NGF), vascular endothelial growth factor (VEGF), brain-derived neurotrophic factor (BDNF), glial cell line-derived neurotrophic factor (GDNF), brain-derived growth factor (BDGF), insulin-like growth factors (IGF), fibroblast growth factors (FGF), and ciliary neutrotrophic factor (CNTF) [10], but also interact with tyrosine kinase receptors to modify the neuronal gene expression profile in to promote axonal growth [34]. Second, SCs produce basal lamina components necessary for nerve regeneration, such as fibronectin and laminin, and growth cones utilize these proteins for adhesion to the basal lamina of the endoneurium for regeneration [35]. Third, transplantation of nerve microtissues at the injury site prevented different SC phenotypes from affecting the axonal guidance [36]. There are many studies on the role of motor and sensory pathways as determinants of axon orientation. After nerve injury, motor axons preferentially regenerate down motor, rather than sensory, nerve branches to reach the end targets, known as preferential motor reinnervation [37]. Based on this phenomenon, Brenner [36] et al. also found that the tibial nerve regenerated well, with many restored fibres, in the motor and mixed nerve graft transplantation groups. However, the tibial nerve in the sensory nerve transplantation group revealed clearly poorer regeneration. Their explanation for the possible reason was the absence of motor elements in the pure sensory pathways, and motor nerve grafts represented an environment rich in SCs that promoted the regeneration of motor axons [38]. Therefore, we did not utilize nerve microtissue from sensory grafts to repair defects in the tibial nerve, thus preventing the inhibition of motor axon regeneration. Fourth, autologous SCs transplantation prevents the immunogenicity of transplanted SCs from influencing the outcome of nerve regeneration [10]. Rodŕıguez and co-workers [10] found that autologous SCs transplantation has a higher functional recovery and number of regenerated fibres reaching the distal nerve than transplantation of isologous and syngeneic SCs. Guénard and colleagues [39] reported that syngeneic adult SCs implanted in PAN/PVC channels enhanced nerve regeneration, while heterologous SCs elicited a strong immune response that impeded nerve regeneration. Finally, SCs play a crucial role in controlling the directionality and speed of axon regeneration across the nerve gap [34, 40, 41]. In a model of sciatic nerve transection injury in mice, the regenerating neurites pass the tips of the proximal nerve in only 6 hours after axotomy [42], while the first migrating SCs are observed 4 days following nerve dissection [41]. These phenomena indicate that axon regeneration occurs significantly earlier than SCs migration. However, axons lack directionality in the early stage of regeneration [41]. After nerve injury, hypoxia within the bridge is selectively sensed by macrophages, which secrete VEGF-A to induce the formation of polarized vasculature in the bridge area [40]. SCs then use the blood vessels as ‘tracks’ to migrate within the bridge on day 4 and overtake regenerating axons on day 5 [40]. The regenerating axons then begin to attach to the migrating SCs on day 6 and follow their trajectories through the nerve gap [40, 41]. These observations not only further suggest that SCs play a crucial role in guiding axon regeneration across the nerve gap, but also reveal that the lack of SCs guidance is the primary reason for axon misdirection in the nerve bridge. Therefore, we can improve the efficiency of the nerve regeneration process by encouraging SCs entry into the bridge to provide a more favourable environment for axonal regeneration.

Our results also suggested that PRP is another possible vein graft filler material that promotes peripheral nerve regeneration and prevents vein collapse. Compared with the Hollow group, the application of PRP improved the proliferation, migration and neurotrophic factor production of SCs, improved electrophysiological parameters, increased the speed and number of regenerated axons, alleviated muscle atrophy and promoted functional recovery. Therefore, after peripheral nerve injury, methods to increase the proliferation and migration of SCs and enhance neurotrophic factor secretion and neurite induction by stimulating SCs, may be important for accelerating the repair of damaged peripheral nerve tissue. PRP, which contains various growth factors that are associated with peripheral nerve regeneration, can play the above roles in different ways. The factors mainly include NGF, VEGF, IGF, platelet-derived growth factor (PDGF), transforming growth factor-β (TGF-β), FGF, and epidermal growth factor (EGF) [43]. Multiple studies have confirmed that NGF/ p75^NTR^-medicated signalling is related to SC proliferation, myelination, and synaptic plasticity [44, 45]. Additionally, administration of exogenous NGF could activate autophagy in dedifferentiated SCs, accelerate the clearance and phagocytosis of myelin debris, and promote the regeneration of axons and myelin sheath in the early stage of peripheral nerve injury [46]. VEGF is a potent angiogenic factor or neurotrophic factor, that can not only stimulate angiogenesis and provide directions for the migration of SCs, but also promote neurite outgrowth and SC proliferation and activate the FLK-1 pathway to enhance nerve survival [40, 47] (Fig. 18). IGF stimulates nerve regeneration by promoting the synthesis of proteins and lipids necessary for nerve regeneration [48]. TGF-b, PDGF, and FGF act as mitogens of SCs [49]. Moreover, PDGF and IGF-1, among the growth factors in PRP, strongly stimulate both the proliferation and migration of SCs by activating the p38 MAPK pathway, and ERK1/2 and PI3K/Akt pathways, respectively [50, 51]. Therefore, PRP may be useful as an emergent coadjuvant therapy to assist the repair of peripheral nerve injury.

Some studies have reported the positive effect of PRP on nerve regeneration [43, 52]. However, most of those studies merely sprayed PRP over the nerve anastomoses [53, 54]. Despite positive results from those studies, simply spraying PRP may waste efficient growth factors. To overcome this limitation, we injected PRP into the lumen of the vein, which served as a PRP reservoir, allowing sufficient time for the release of growth factors. Platelets begin to release approximately 95% of the pre-synthesized growth factors within 1 hour after activation [55], and the remaining growth factors are gradually synthesized and released over the next 5 to 28 days [55]. Thus, PRP may accelerate the regeneration of axons and blood vessels in the early stage of nerve repair due to the influence of rich neurotrophic factors. This hypothesis may be reflected in the results of this study. Early postoperative CEUS showed that blood perfusion and VEGF mRNA expression in grafts were significantly higher in the PRP group than in the Hollow group, and the regeneration axon density at 4 weeks was also significantly higher than that in the Hollow group. Ye [56] et al. reported that PRP combined with SCs as the filler for poly (lactic-co-glycolic acid) conduits can significantly increase the number of regenerated axons and myelin sheath thickness and improve composite muscle action potential and nerve conduction velocity. PRP has also been reported as an effective filling material for artificial or vein grafts due to its role in elongating the site of the regenerating axons [13, 57]. Therefore, the above results all demonstrate the effectiveness of PRP, which is consistent with the results of our study.

PRP was prepared from autologous blood sampling in our study, which has the advantages of a low risk of immunological side effects and is rapid, simple, convenient, and economical. The role of WBCs present in PRP in nerve regeneration or tissue healing remains controversial [58]. Although WBCs may have valuable anti-inflammatory effects, they may impede the nerve regeneration process through the production of bioactive catabolic cytokines [59]. Thus, PRP with a relatively low leucocyte concentration was prepared in this study.

We also found that the Micro-T + PRP group showed the stronger proliferation and migration responses of SCs and faster axonal regeneration of DRGs than the Hollow, PRP and Micro-T groups. Meanwhile, the levels of the Micro-T + PRP and Autograft groups were similar in the neurological function recovery, positive electrophysiological response, ultrasonic detections and histomorphological changes (including increased axon, myelin sheath and improved collagen fibre areas). These results may be explained by the combined advantage of SCs interacting with PRP in promoting nerve regeneration. In addition, the results could account for the significant increase in the number of microvessels (VEGF mRNA expression, 2 weeks) and axons (NF-200 staining, 4 weeks) in the Micro-T + PRP group at the early stage of treatment. Combined with activated PRP gel, nerve microtissues did not leak outside of the venous conduit, allowing sufficient time for nerve repair in the venous conduit. Hence, novel nerve grafts constructed by nerve microtissues and PRP in conjunction with autologous veins may be more effective in promoting peripheral nerve regeneration.

Neuromuscular ultrasound, a highly sensitive technique, is becoming a preferred imaging technique for the evaluation of peripheral nerve and muscle diseases [60, 61]. When performed simultaneously with nerve conduction studies, it provides dynamic and structural information that can refine the diagnosis or help to determine the structural aetiology [61]. In the present study, routine high-frequency ultrasound enabled assessment of the localization, continuity, echogenicity and lumen thickness of vein grafts, as well as the targeted muscle echogenicity and morphological aspects, such as muscle fatty degeneration or atrophy, but it was not sufficient to comprehensively evaluate nerve regeneration. Therefore, the detection of peripheral nerve repair by new multimodal ultrasound new techniques, such as CEUS, SWE and AngioPLUS, is essential.

Few studies have used CEUS to study vasa nervorum and nerve tissue perfusion. CEUS uses lipid microspheres, which are gas-filled microbubbles (2–5 μm in diameter) that can generate signals detected by ultrasound and can directly visualize blood flow at a capillary level two-dimensionally in real time [62]. It is well-accepted that angiogenesis and nerve regeneration are intimately connected processes. For example, Ferretti et al. [63] showed that vascularization precedes the process of innervation in the transplanted human skin equivalent model. Cattin et al. [40] confirmed that macrophages within the bridge secrete VEGF-A after nerve dissection, which stimulates the formation of blood vessels, and the SC cords use the polarized blood vessels as a migratory scaffold to guide axons to enter and cross the bridge. These results indicate that the development of nervous tissue is driven by the vascular system. As a result, we attempted to use CEUS examination to assess microvessel perfusion of the regenerative nerve.

In our CEUS examination, the Micro-T + PRP and Autograft groups (no significant differences between these groups) showed a significant improvement in the microvascular blood perfusion of the regenerating nerve at 2 weeks after surgery, as reflected by the TTP, PI and AUC values. Meanwhile, the AUC was positively correlated with VEGF mRNA expression. Histological results also further demonstrated that nerve regeneration in the Micro-T + PRP group was significantly superior to that in the other vein graft groups. Consequently, CEUS may become an imaging evaluation method with which to assess the process and prognosis of nerve repair.

Within the musculoskeletal system, CEUS has also recently been applied to determine functional dynamic perfusion patterns in muscle tissue, beyond morphological aspects such as muscle fatty degeneration or atrophy that are routinely detected by ultrasound [64]. As reported, blood flow is closely related to skeletal muscle activity, and the absence of the VEGF gene leads to a substantial reduction in muscle capillarity, while neural factors play an important role in maintaining the expression of VEGF in limbs and reducing the degree of limb ischaemia [65]. Thus, once the targeted muscle is reinnervated by regenerated nerves, the VEGF signalling pathway may increase, and possibly increasing the blood supply to the muscle [65]. Hence, we speculated that the abundant muscle blood perfusion in the Micro-T + PRP group may be attributed to the fact that the regenerated nerves reinnervate the activity of targeted muscle, upregulate VEGF expression, stimulate angiogenesis, and improve vascular permeability, suggesting that CEUS examination of the target muscle can also provide useful information for the prognosis of peripheral nerve injury.

SWE is an imaging technology sensitive to tissue stiffness that has received substantial attention in recent years in the non-invasive assessment of tissue mechanical properties [66]. While ultrasound elastography has shown promising results in the non-invasive assessment of liver fibrosis, breast tissue, the thyroid, the prostate, and lymph nodes, new applications in neuromuscular diseases are emerging [66]. For instance, Lacourpaille and collaborators [67] found that the stiffness of all examined skeletal muscles was significantly higher in patients with Duchenne muscular dystrophy than in healthy controls. Shear-wave velocities decreased with increasing fat content in the supraspinatus muscle in patients with rotator cuff disease [68]. Quantitative measurements of targeted muscle elasticity in the present study showed that Young’s modulus was positively linearly correlated with the percentage of collagen area in the muscle. The less stiff the muscle was, the lower the elastic modulus, suggesting a lower degree of tissue fibrosis and better repair of muscle tissue. Muscle stiffness in the Micro-T + PRP group was significantly lower than that in the Hollow, PRP and Micro-T groups, indicating that SWE examination may be an effective method for monitoring prognosis.

Recently, a new and innovative paradigm has been developed in colour Doppler performance that overcomes the limitations of conventional Doppler US for microvessel evaluation1, called AngioPLUS (PlaneWave UltraSensitive Imaging; SuperSonic Imagine, Aix-en-Provence, France) [18, 69]. AngioPLUS can be used in SuperSonic Imagine’s Aixplorer ultrasound system. By using unfocused or plane waves and 3-dimensional wall filtering techniques, it enhances the sensitivity and resolution of microvascular flow detection, thus providing much more detailed information for visualizing and detecting slow blood flow in microvessels [18]. In our study, 12 weeks after the operation, the stiffness of the target muscle was negatively correlated with the microvascular flow density, indicating that the higher the degree of muscle fibrosis, the fewer regenerated microvessels in the muscle, which was consistent with the results of CEUS examination of the muscle. Hence, AngioPLUS may be used as a microvascular flow detection technique comparable to CEUS but more convenient than CEUS.

Consequently, multimodal ultrasound, as a convenient and noninvasive detection method, may become a promising multidimensional imaging evaluation method for assessing the process and prognosis of peripheral nerve injury.

This study has several limitations. First, vein grafts repaire nerve defects of only 12-mm in length, and the feasibility of repairing a long-distance defects needs to be further studied. Second, few studies have used nerve microtissues combined with PRP to repair peripheral nerve defects; thus, there is a lack of relevant and comparable studies. Furthermore, the ultrasound examination of peripheral neuropathy has many chanllenges, depending, for example, on the size and depth of the nerve and the proficiency of the operator. Thus, experienced radiologists are required to participate in the research of this type.

## Conclusion

In conclusion, a novel tissue-engineered nerve graft composed of an autogenous vein, nerve microtissues and PRP showed excellent efficacy in repairing 12-mm defects of the tibial nerve in rabbits. Moreover, multimodal ultrasound (high-frequency ultrasound, SWE, CEUS and AngioPLUS) may provide a clinical reference for prognosis by allowing the stiffness and microvascular flow of nerve grafts and target muscles to be evaluated quantitatively.

## Data Availability

Not applicable.
